# Vision and air flow combine to streamline flying honeybees

**DOI:** 10.1038/srep02614

**Published:** 2013-09-10

**Authors:** Gavin J. Taylor, Tien Luu, David Ball, Mandyam V. Srinivasan

**Affiliations:** 1 Queensland Brain Institute, The University of Queensland. Brisbane, QLD, 4072, Australia; 2 ARC Centre of Excellence in Vision Science, The Australian National University. Canberra, ACT, 0200, Australia; 3 School of Electrical Engineering and Computer Science, Queensland University of Technology. Brisbane, QLD, 4000, Australia; 4 School of Information Technology and Electrical Engineering, The University of Queensland

## Abstract

Insects face the challenge of integrating multi-sensory information to control their flight. Here we study a ‘streamlining' response in honeybees, whereby honeybees raise their abdomen to reduce drag. We find that this response, which was recently reported to be mediated by optic flow, is also strongly modulated by the presence of air flow simulating a head wind. The Johnston's organs in the antennae were found to play a role in the measurement of the air speed that is used to control the streamlining response. The response to a combination of visual motion and wind is complex and can be explained by a model that incorporates a non-linear combination of the two stimuli. The use of visual and mechanosensory cues increases the strength of the streamlining response when the stimuli are present concurrently. We propose this multisensory integration will make the response more robust to transient disturbances in either modality.

An insect in flight has available to it at least two sources of information about the speed of its flight through the environment. One source is the visual perception of motion through the world, derived from the pattern of image movement (optic flow) as sensed by the visual system. The second source is the movement of air over the body. Individually, these cues can be unreliable for inferring the insect's own motion in the environment, because variations in the topography of the environment, movement of objects in the world, or a gust of wind can create erroneous or conflicting sensory cues.

Many insects use visual information to control their flight. An example is the optomotor response, which allows a flying insect to correct unwanted rotations about its body axes by generating turning responses that compensate for the rotational optic flow that it senses^1^. Other aspects of flight are also visually controlled: for example, honeybees^2^ and *Drosophila*^3^ have been shown to use visual cues to regulate their flight speed, to avoid dangerously close objects (honeybees^4^, *Drosophila*^5^), to orchestrate safe landings^6^ and to centre their flight through a narrow passage^7^. Honeybees have also recently been shown to use the rate of visual motion to control their abdominal posture^8^. The faster the visual motion that is experienced by the yes, the greater the elevation of the abdomen. This behaviour, termed the ‘streamlining response', should minimise the aerodynamic drag experienced by the insect by reducing the cross-sectional area of its body that is exposed to the wind^9^. However, it is not yet known whether this streamlining response is mediated purely by optic flow, or by additional cues such as the air flow that is experienced during flight.

Vision is not the only sense that some insects use to stabilise their flight. The hindwings of Diptera have evolved into specialised club shaped masses, called halteres, which oscillate in time with the wingbeat^10^. These structures provide flies with a gyroscopic sense for the detection of unintended rotations, complementing their visually evoked optomotor response^11^. The antennae of *Manduca* were recently shown to sense gyroscopic forces in a similar way to dipteran halteres^12^.

Many experiments have shown that visual and mechanosensory cues could stabilize insect's flight *against* external disturbances caused by air movements in the environment. For example, if a gust of wind blows an insect off course, the resulting optomotor response should help correct the unintended deviation from the flight path^13^. Honeybees flying down a corridor were found to be able to regulate their ground speed in the presence of strong head wind^14^. This compensation was accomplished by holding constant the perceived optic flow^14^.

However, despite some reflexes that seemingly counteract the unintended disturbances caused by air movements on an insect's flight plan, air speed is also actively sensed and used to control other aspects of flight. Many insects, including honeybees^15^, have also been shown to sense air speed and to use this to control the amplitude of their wingbeat, which may act to regulate their flight speed (for a full review see Taylor and Krapp^16^). Other insects sense air movements to detect changes in flight direction. For example locusts^17^,^18^, *Drosophila*^19^, and carrion beetles^20^ respond to air flow by changing the direction of their path in a compensatory fashion.

It appears, therefore, that insects are able to make use of air flow as well as visual cues to control various aspects of their flight. To investigate the effects of air flow on the control of the honeybee's streamlining response, we exposed tethered honeybees to combinations of optic flow and air flow in a flight arena, and observed the reactions of the abdomen. We also examined whether the antennae contribute to the measurement of air speed, and developed a quantitative model that describes how this is accomplished.

## Results

**Figure 1:**
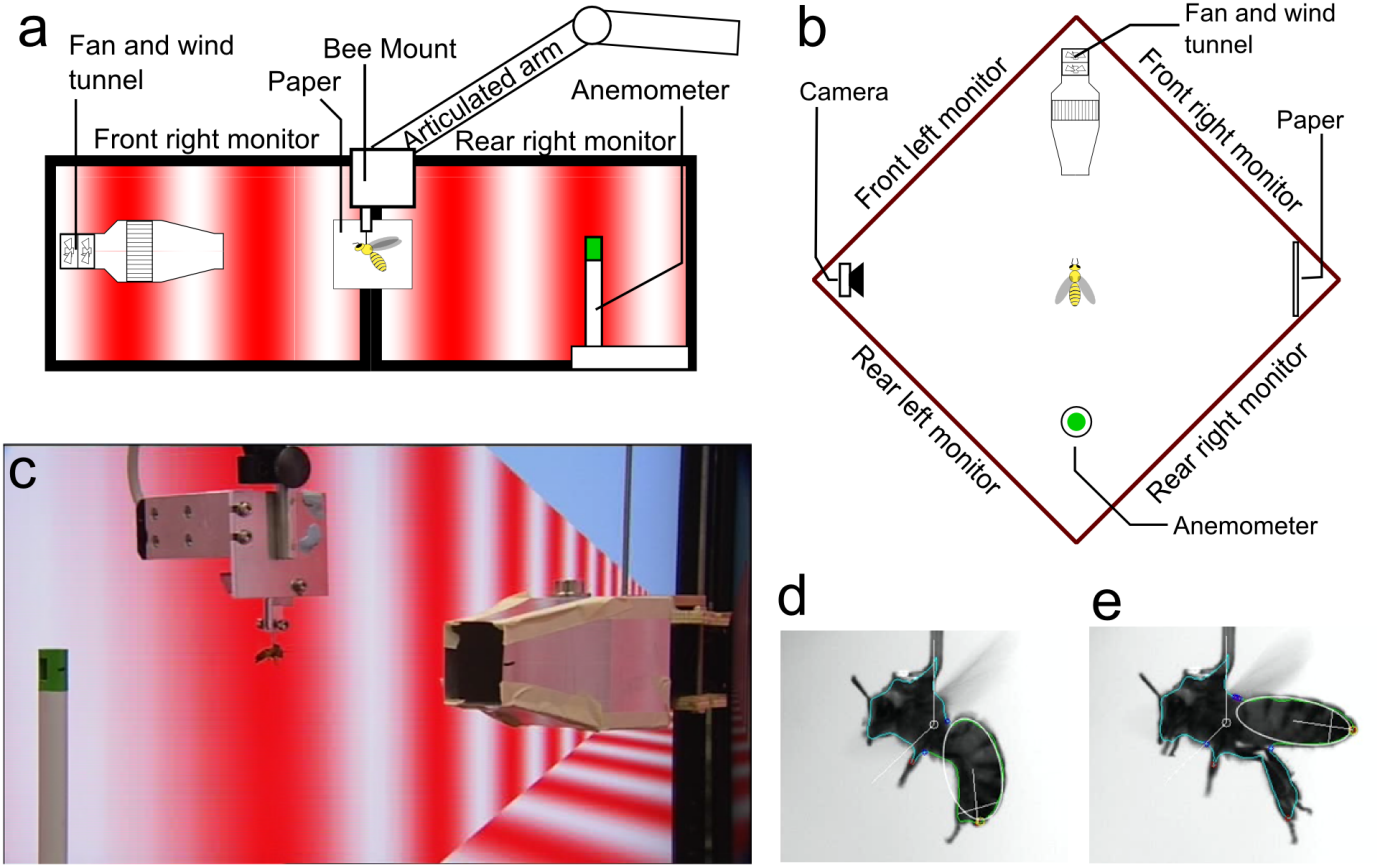
Overview of the tethered flight arena. (a): Schematic side view of flight arena, from the camera's perspective, (b): Schematic top view of flight arena (bees are not to scale), (c): Photo of a tethered honeybee in flight taken from the position of the rear right monitor, (d): Image of honeybee with a lowered abdomen, (e): Image of honeybee with a raised abdomen. (d) and (e) are representative of video images used for analysis. The white ellipses depict the results of automatic image-based segmentation of the insect's abdomen and determination of its orientation, as described in ‘Methods'.

The effect of combined air speed and optic flow stimulation on the orientation of the honeybee's abdomen was investigated by placing tethered honeybees in a flight arena (Figure 1a, b and c), based on the design of Luu et al.^8^. A near-panoramic array of computer monitors visually simulated flight along an infinitely long tunnel, and a fan in front of the insect generated a headwind at a controllable velocity. The behavioural response of the tethered insect was recorded using a video camera placed at the side of the arena, and was digitised automatically in real time. Honeybees were exposed to optic flow at speeds ranging from 100–600 deg/s in 100 deg/s steps, at a constant air speed which varied from 0–5 m/s across different experiments.

Luu et al.^8^ found that honeybees did not exhibit a streamlining response when no visual motion was displayed (0 deg/s optic flow). Our initial observations confirmed that, regardless of air speed, bees would not fly reliably nor hold a stable abdomen position when no visual motion was shown in the front-to-back direction. (Occasionally, the abdomen would be raised at the onset of flight, but would then drop to a non-streamlined position, after which flight would cease). Because of this, we did not include the speed of 0 deg/s in our optic flow test protocol. The experimental apparatus and protocol are described in greater detail in the ‘Methods' section.

### Abdominal response to a combination of air speed and optic flow stimuli

**Figure 2:**
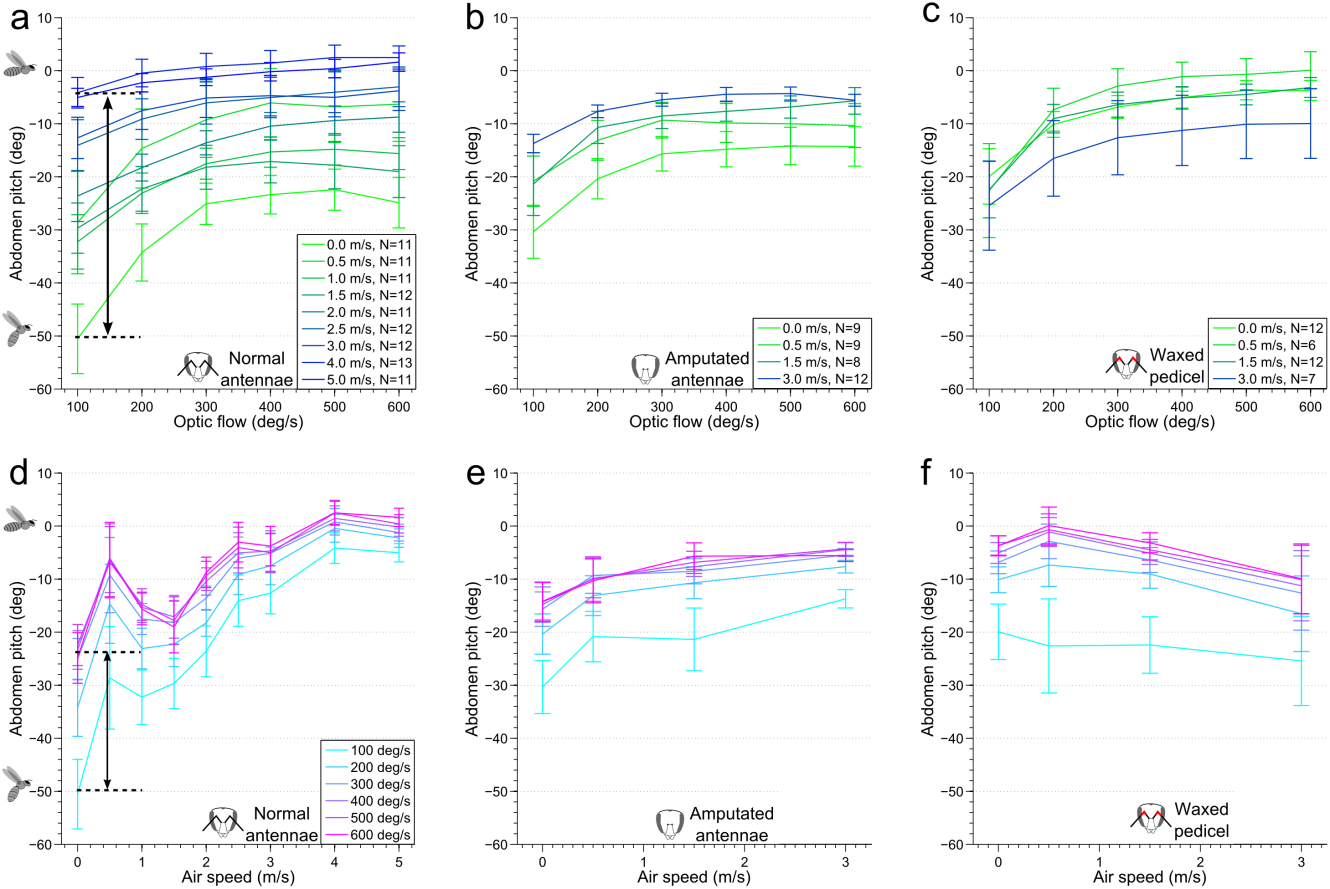
Abdomen response in honeybees depends on optic flow and air speed. Plotted as a function of optic flow with air speed as a parameter (a), (b), (c), and as a function of air speed with optic flow as a parameter (d), (e), (f). Note that honeybees would not fly reliably at 0 deg/s optic flow, hence this data point is omitted. (a, d) represent data from intact bees, (b), (e) data from bees with amputated antennae, and (c), (f) data from bees with waxed pedicels. The legends in (a, b, c) show the sample size of bees tested at each airspeed, for a particular antenna condition. The legend in (d) is used for (e) and (f). Error bars show ± s.e.m.

In a first set of experiments, we investigated the strength of the streamlining response that was evoked by various headwind speeds, and various velocities of optic flow. These experiments revealed that, in addition to visual motion, air flow plays an important role in driving the honeybee's streamlining response. Tethered honeybees flying in the arena display their characteristic streamlining response to optic flow, as described in Luu et al.^8^. We find, however, that this visually induced response is modulated by wind. Specifically, the range of the visually evoked response decreases as the air speed is increased (Figure 2a). The reason for this is that when the insect is stimulated with air flow, the abdomen is generally raised further, having the overall effect of making the animal more streamlined (Figure 2d). The variation of the response with air speed for any fixed velocity of the visual stimulus (Figure 2d) indicates that the abdomen angle does not increase monotonically with air speed - it shows both a local minimum and a maximum before plateauing. Air speed appears to account for a greater range of the response than optic flow: at low optic flow (100 deg/s), the abdomen pitch varies over a range of ~ 45° in response to variation of air speed (Figure 2a, black arrow). In contrast, in the ‘no wind' condition the abdomen pitch changes only over a range of ~ 25° in response to the variation of optic flow (Figure 2d, black arrow).

Statistical analysis of the data in Figure 2(a and d) using ANOVA (Supp. 7.a) showed a significant effect of optic flow (F_1.6,147.3_ = 141.39, *p* < 0.000001), as well as air speed (F_8,95_ = 6.35, *p* = 0.000001), and an interaction between the two variables (F_12.4,147.3_ = 4.89, *p* = 0.000001) on abdominal pitch. Figure 2a shows that as the air speed increases, the dependence of the response on optic flow decreases (explaining the interaction effect observed), however, regardless of optic flow level, the response shows a strong dependence on air speed (Figure 2d). Beyond 400 deg/s, post-hoc tests show that there is no significant difference in abdomen position, confirming that the response has indeed saturated (this saturation level varies between 300 and 500 deg/s for the antennal manipulation cases described in the following section, but is qualitatively similar across all antenna conditions, (Supp. 7.a, b and c)). Air speeds are divided into four different groups by post-hoc tests. First, the global response minimum occurs in the ‘no wind' condition. This is followed by a local maximum at 0.5 m/s (the response of this depends heavily on the optic flow interaction), which is succeeded by a local minimum centered at 1.5 m/s, before the response plateaus beyond 2.5 m/s.

In a second set of experiments we investigated the effects of a tail wind. When exposed to a negative air speed, simulating a tail wind, honeybees exhibit a reduced response to air speed, although optic flow continues to have a similar effect. Details of the streamlining response evoked by various strengths of tail wind and various speeds of optic flow are given in Supp. 1.

The pitch of the thorax of a freely flying bee changes, depending on its air speed^21^. Therefore it is of interest to investigate whether, and if so how the streamlining response varies with thorax pitch. Accordingly, we measured the streamlining response when the bee was tethered with its abdomen oriented at various pitch angles. The results, described in Supp. 3, show that there is no significant difference in the streamlining response elicited by visual motion or air flow, between bees that are tethered with their thorax oriented for fast flight (at a low thorax pitch of 0°) and bees that are tethered with their thorax oriented for hover (at a high thorax pitch of 36°). Because thoracic reorientation also rotates the honeybees head, this implies the streamlining response is invariant to the perceived direction of optic flow and air movement (and also the direction of gravity) for head orientation angles between 0° and 36°. However, tethering with the thorax pitched downwards (−33°) does produce significant changes in the streamlining response (Figure S3).

Finally, it is possible that aerodynamic forces may act to passively raise the abdomen into a streamlined position. We tested this using euthanized honeybee bodies positioned in air streams up to 4 m/s and observed minimal change in abdomen position across the range of air speeds (Supp. 2). This indicates the streamlining response to air speed is a primarily active response.

### Manipulation of the antennae

**Figure 3:**
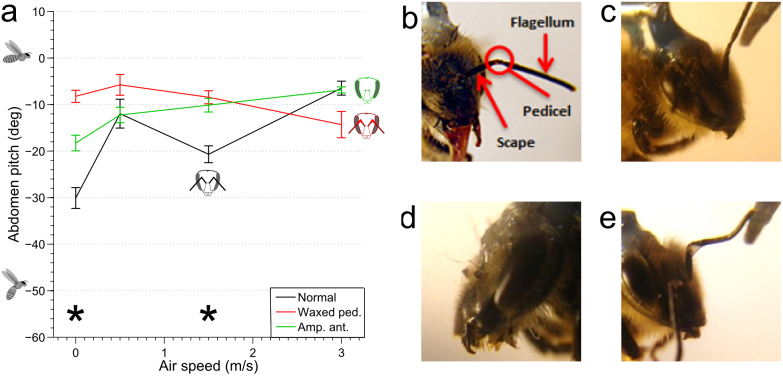
Comparison of honeybee's abdomen response across various experimental conditions. (a): Comparison showing the mean response across all optic flow levels at each air speed, for all conditions. *Denotes a significant difference between normal bees and both manipulations at the indicated air speed. Error bars show ± s.e.m. The sample size for each air speed and antenna condition are present in the legends in Figure 2(a), (b), (c). (b): Antenna morphology, (c): Flexibility of non-waxed antenna, (d): Amputated antennae, (e): Illustration of immobilisation and lack of flexibility of waxed pedicel joint.

The antennae, and specifically the Johnston's organs (the mechanosensors that detect movement of the flagellum^22^) located in the antenna's pedicle joint (Figure 3f), have previously been reported to provide honeybees^15^ and other insects^16^ with a measurement of air flow in flight. To examine whether these receptors provide the honeybee with information to regulate its flight posture, we performed two manipulation experiments, firstly, amputation of the antenna (Figure 2b and e), and, secondly, immobilization of the antennal pedicle with wax (Figure 2c and f). The abdominal responses of manipulated insects, exposed to three different air speeds: 0.5, 1.5 and 3 m/s, as well as to the ‘no wind' condition, were measured for comparison with the intact controls. This reduced set of air speeds were selected as the points of interest from the responses of the un-manipulated honeybees to air flow, as they represented the global minimum (0 m/s), the local minima (1.5 m/s), the local maximum (0.5 m/s), and a point well into the saturated region of the response curve (3 m/s). We hypothesized that if the antennae were responsible for the measurement of air speed, then there would be a difference between the responses of the treated animals and the untreated controls, especially at these points of interest in the original curve.

Neither the amputation of the antenna, nor waxing the pedicle appeared to affect the basic characteristics of the honeybee's response to optic flow – the response continued to increase monotonically with optic flow (Figure 2b and c). Unexpectedly, however, at low to intermediate air speeds (0, 0.5 and 1.5 m/s) the response versus optic flow profiles of the manipulated honeybees were generally *higher* than those of the controls. Furthermore the local minimum that is clearly present at an air speed of 1.5 m/s in the responses of the control animals (Figure 3a) was no longer evident in the manipulated animals. ANOVA tests (Supp. 7.b and c) to analyse the influence of air speed on abdomen pitch showed no significant effect in the case of the waxed pedicels (F_3,33_ = 0.72, *p* = 0.546), and a weak effect in the case of the amputated antennae (F_3,34_ = 3.11, *p* = 0.039). Thus, the change in abdomen position in response to wind is removed or reduced in the manipulated bees. In both types of manipulation, the effect of optic flow remained similar to non-manipulated bees, whilst the interaction between air speed and optic flow was removed. This indicates that antennal manipulation removes or reduces wind-induced variation of the response.

When comparing normal honeybees with the groups of bees that had been subjected to the two kinds of antennal manipulation, we found a significant effect of antennal manipulation at 0 and 1.5 m/s air speed, but not at 0.5 and 3 m/s air speed (Figure 3e). Post-hoc testing showed that both antennal manipulation conditions were significantly different from the control at the former air speeds (Supp. 7.d). The reason for these differences in the manipulated animals, is that the responses in the no-wind condition and at 1.5 m/s are stronger than in the intact controls. This finding implies, surprisingly, that under no-wind conditions and at intermediate air speeds, the input from the antennae *inhibits* the abdominal pitch that is observed in normal honeybees.

### A model of the interaction between air speed and optic flow

Previous investigations of other flight control behaviours that are driven by visual as well as mechanosensory stimuli in *Drosophila*^11^ and *Manduca*^23^ have documented multimodal responses that can be accurately characterized as a linear, or weighted linear, summation of the visual and mechanosensory components of the response.

**Figure 4:**
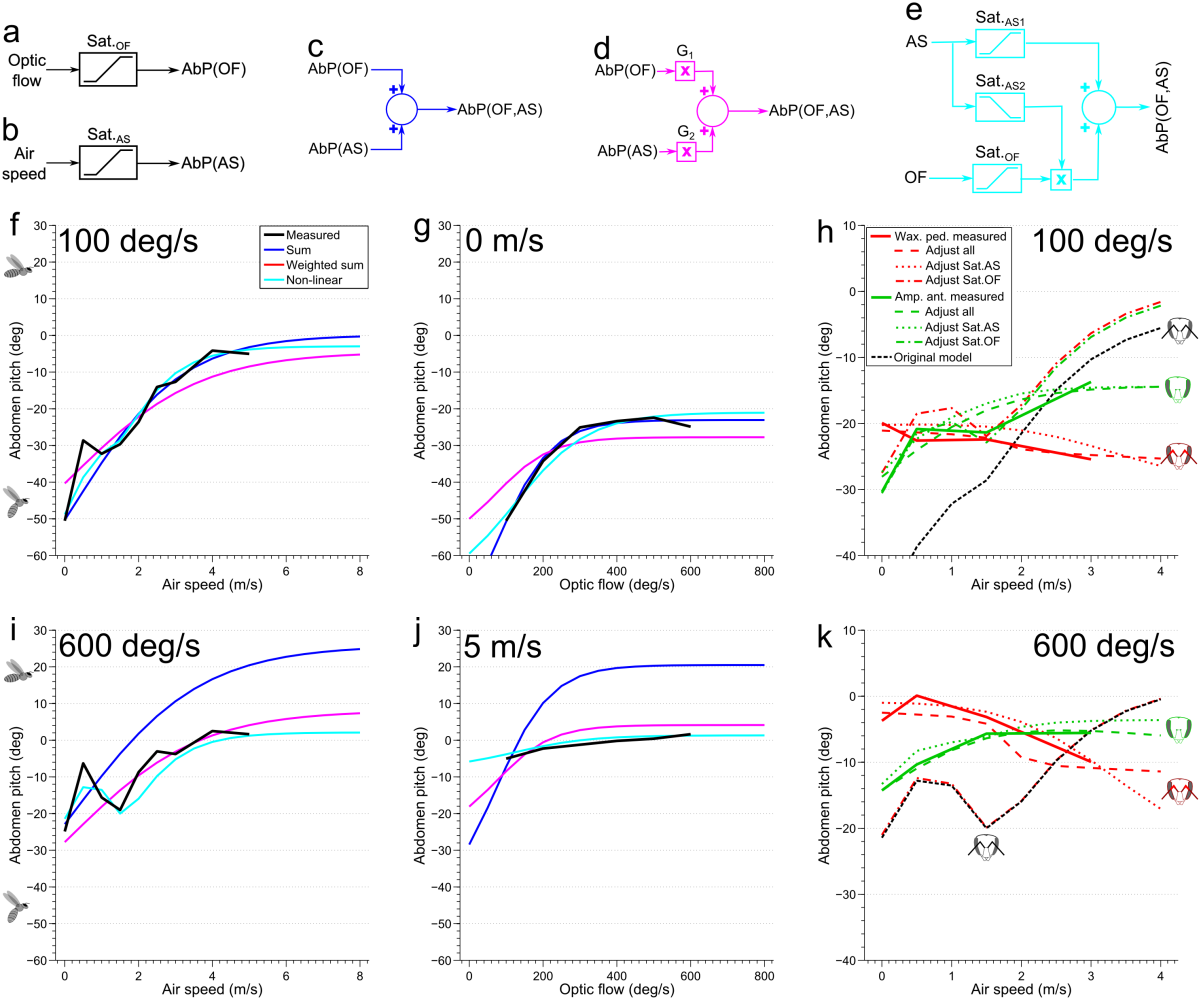
Model predictions of the abdomen pitch. Model types shown: (a), (b) Abdomen pitch response as a function of each stimulus, (c) Linear summation, (d) Linear weighted summation, (e) Non-linear combination. (f), (g), (i), (j) Response comparisons for all models at the boundaries of the measured response surface: (f) Response vs. air speed at 100 deg/s optic flow (the legend in this plot is used for (f), (g), (i), (j)), (g) Response vs. optic flow at 0 m/s air speed, (i) Response vs. air speed at 600 deg/s optic flow, (j) Response vs. optic flow at 5 m/s air speed. (h), (k) Response comparison of non-linear model refit to data from antenna manipulated bees, at low and high optic flow boundaries of the response surface (Model details and plots at the low and high air speed boundaries are shown in Supp. 5.b). Some model parameters are refit (separately for each type of antennal manipulation), whilst others are held at the same values as those found for non-manipulated bees: (h) Response vs. air speed at 100 deg/s optic flow (the legend in this plot is used for (h), (k)), (k) Response vs. air speed at 600 deg/s (note that the curve for Adjust Sat.OF for both manipulations overlap the curve for the original model). Abbreviations: AS – air speed, OF – optic flow, AbP – Abdomen pitch, G – Gain. Details of all model parameters are given in Supp. 5.

We examined whether the abdomen response to the optic flow or to the wind could each be described by an input-output relationship that involved a saturating nonlinearity (Figure 4a and b). It was found that, in each case, the input versus output data were well fitted by a variable slope sigmoidal equation^24^, using least squared nonlinear regression (Figure 4f and g). Details of all models are given in Supp. 5.

How do optic flow and wind interact to generate the streamlining response? If the net response is a linear summation of the individual response to each stimulus, then it should be possible to predict the response to the combined stimuli by summing the (saturating) response to each stimulus. For example, it should be possible to predict the response to optic flow at 100 deg/s and an air speed of 5 m/s from the response to an optic flow at 100 deg/s (with zero air speed), and the response to wind at 5 m/s (with zero optic flow). However, when the two saturating responses are combined as a linear summation (Figure 4c), the result substantially overshoots the measured response for combinations of the two stimuli (Figure 4i and j).

A weighted sum of the two original saturating responses was also tested (Figure 4d). The optimal weightings for both saturation functions were found using a least squares fit across the entire response surface; optic flow was weighted by a factor of 0.45 and air speed by a factor of 0.74 (Figure 4d, G_1_ and G_2_ respectively). Whilst this weighted sum was found to perform well at predicting the air speed response curve for low and high optic flows (Figure 4f and i), it was less successful at predicting the entire response versus optic flow curve. Notably, the *range* of the optic flow response decreases between the 0 and 5 m/s air speed conditions, which is not captured by the weighted sum model (Figure 4g and j).

**Figure 5:**
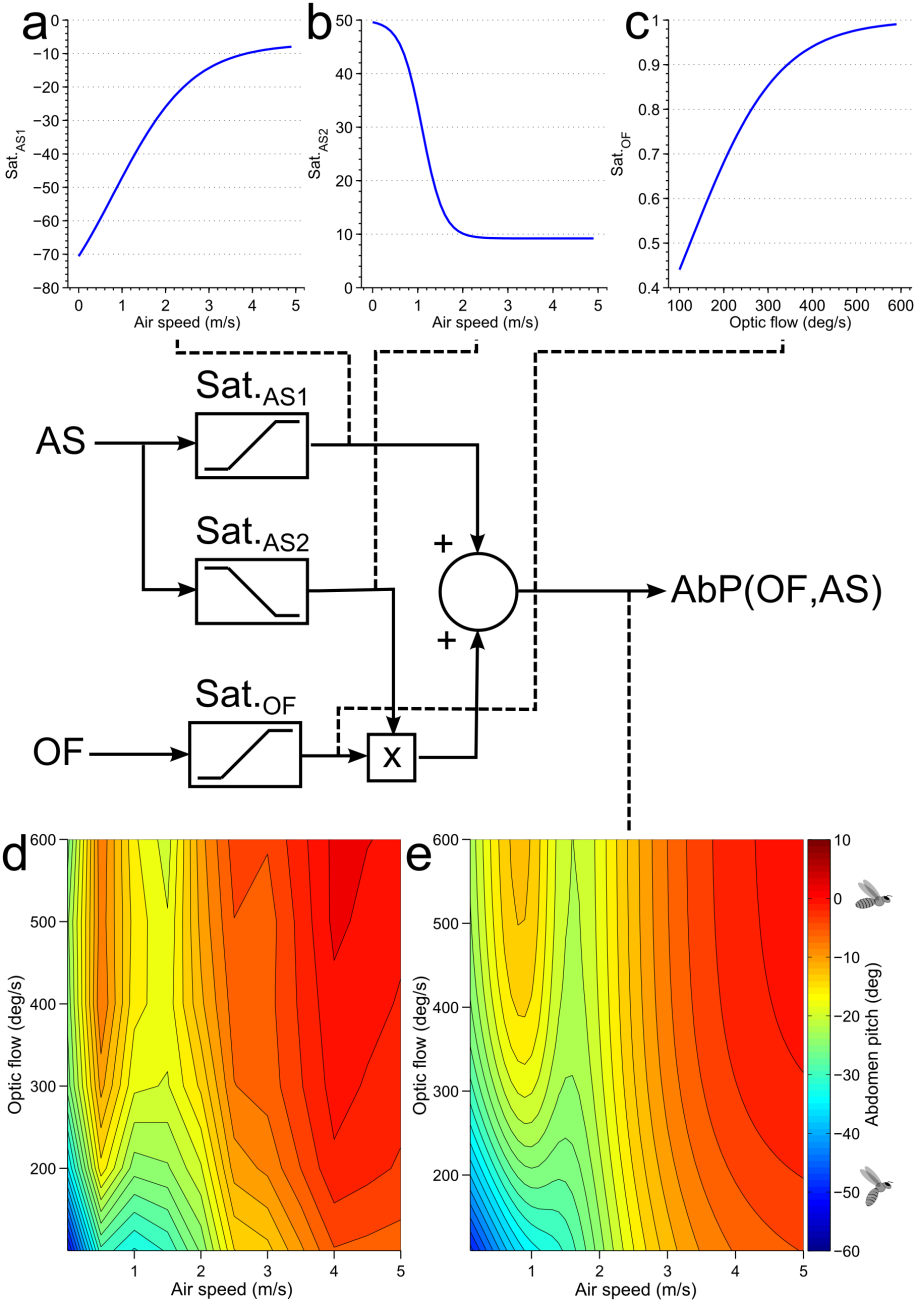
Model describing the non-linear interaction of air speed and optic flow. The plots indicated by dashed lines show the input-output relationships at various stages of the model. (a) Response to air speed, (b) Modulation of gain of the optic flow path way by air speed, (c) Response to optic flow, (d) Measured response, (e) Predicted response. Abbreviations: AS – air speed, OF – optic flow, AbP – Abdomen pitch. Model details are shown in Supp. 5.a.

Finally, the response was modeled as a non-linear combination of the two stimuli. Whilst there are many possible ways in which combining these responses could be combined nonlinearly, we found that a linear summation of the saturating response to each of the two stimuli, combined with an interaction in which the magnitude of the response to optic flow was modulated by the air speed (as shown in Figure 4e), was able to predict the observed ranges of the responses to the two stimuli, either in isolation or in combination (see Figure 5d and e). To modulate the optic flow response by air speed, a saturating function of air speed, Sat._AS2_ (Figure 5b) was used to adjust the gain along the optic flow pathway, Sat._OF_ (Figure 5c). While the output of Sat._AS1_ (Figure 5a) increases as the air speed is increased, the output of Sat._AS2_ decreases with increasing airspeed (Figure 5c). This interaction can be viewed as a gain control that is exerted by the airflow sensing pathway on the optic flow sensing pathway, in which the gain of the optic flow pathway is progressively reduced as the air speed increases. This postulated interaction successfully predicts the peak that is consistently observed at 0.5 m/s in the response to variation of air speed (Figure 4i), which arises from slight differences in the thresholds and slopes of the saturating functions Sat._AS1_ and Sat._AS2_.

A summary of the model, showing the block diagram and the exact profiles of the three nonlinearities is shown in Figure 5. This figure also shows a comparison of the experimentally measured two-dimensional response surface (Figure 5d) with that predicted by the model (Figure 5e). This model is designed to predict the response for positive air speeds and optic flows, the standard flight conditions for a honeybee.

### Streamlining in the absence of antennal information

Whilst this model captures the response of normally tethered honeybees, can one predict the honeybee's streamlining responses when they are deprived of air flow information from their antennae? From Figures 2 and 3 it is clear that whilst both antennal manipulations are significantly different from the control case, the response is also modulated by air speed to some extent, which in the case of antenna amputated bees is significant. From this, we might assume that streamlining is not only driven by optic flow following these manipulations, but that the honeybee receives some measure of air speed from other sensory organs, that would usually be combined with information from the antenna.

If all model parameters are refit (Supp. 5.b), then the model is able to predict the honeybee's response to air speed after either type of antennal manipulations (Figure 4h and k, and Figure S6). Notably, different model fits are required to capture the observation that streamlining is increased at high air speed when the antennae are amputated, but decreased at similar air speeds when the pedicels are waxed. It turns out that in both cases the response range, or gain, of both saturating functions is reduced (Table S2-1), which might be expected given that the honeybee's primary air speed sensor has been disabled. However, it is surprising that honeybees react differently to the two different manipulation cases, given that the same sensory information has been removed (waxing a honeybee's pedicel has been shown to completely remove any response to air speed from the antennal nerve, which would otherwise be present in intact antennae^15^). In both cases, they would presumably have the same remaining alternate, but unknown, mechanisms of sensing air speed. If the sensory systems are the same and the outputs are different, but our model can still predict the outcome, then it appears that the manipulated bees must have used different weightings at the neural level for combining the two sensory modalities. We shall comment on this further in the Discussion.

To further elucidate how honeybees made adaptations to use what air speed sense they had remaining after antennal manipulation, we tested if some model parameters could be held constant, at the level that fit the data for normal honeybees, whilst others were refit. In brief, if only the parameters in both saturating functions to air speed (Sat._AS1_ and Sat._AS2_) are allowed to vary, the model can be fit nearly as well as when all parameters are adjusted. Conversely, if only the parameters in the saturating response to optic flow (Sat._OF_) are allowed to vary, the fit is qualitatively no better than the unadjusted model (Figure 4h and k and Figure S6). Further, if the terms in only one of Sat._AS1_ or Sat._AS2_ are recalculated, relatively good model fits are achievable, but they fail to capture the decrease in abdomen pitch at high air speeds and low optic flow rates for pedicel waxed bees (Figure S6). Nevertheless, the model captures most of the observed responses, particularly for the antennal amputation. In all cases, there is considerable flexibility in using this model to explain the behaviors that are observed under all of the experimental conditions.

## Discussion

Our findings show that honeybees measure both air speed and optic flow to actively control their abdominal angle during tethered flight. The response to wind is asymmetrical, showing that honeybees differentiate between head and tail winds. This is in concordance with the effect of optic flow, which is also asymmetrical; honeybees respond by elevating their abdomen to progressive optic flow but not to regressive flow^8^. Given the relatively small movements of the abdomen that are observed in tethered honeybee bodies, it seems likely that the raising of the abdomen is primarily an active response that is driven by sensing optic flow and air flow, rather than passive mechanical lifting of the abdomen by air flow, which has previously been suggested^9^,^21^.

Luu et al.^8^ proposed that the raising of the abdomen serves to actively streamline the animal and reduce its energy consumption during flight. The present study lends support to this idea, as combining information from visual motion and airflow causes the abdomen to be raised into increasingly streamlined positions, further contributing to the energy savings of the insect in flight. The range of the air speed response curve at low optic flow is approximately 1.8 times that of the optic flow response curve with ‘no wind'. In other words, wind appears to be more effective than optic flow at strengthening the streamlining response. The additional use of optic flow may increase the robustness of the response to fluctuations in air speed arising from turbulence in natural flight.

Our study indicates that the abdomen response to combinations of optic flow and air speed involves nonlinear interactions between the two sensory modalities. These interactions cannot be predicted accurately by using a weighted linear summation of the response to either stimulus. This is in contrast to other studies of visual and mechanosensory integration in insects, such as *Drosophila*^11^ and *Manduca*^23^, where multimodal responses have been found to combine as a linear or weighted sum to generate a response. We find that, in the case of the streamlining response, a non-linear combination of the saturating responses to wind and optic flow, where increasing air speed decreases the magnitude of the response to optic flow, allows the model to predict the responses to various combinations of the two stimuli, as well as the unexpected peak at 0.5 m/s. Thus, a relatively simple, nonlinear combination of two stimuli can create an apparently complex behavioral response. Furthermore, this model can also be refit to predict the response after antennal ablations (assuming the honeybees have some other mechanism of sensing air speed), showing the model is capable of predicting some of the effects of manipulating the wind sense.

Our antennal manipulation experiments show that the antennae contribute to regulation of the insect's abdominal position in response to wind. When the antennae are either amputated or the Johnston's organs are immobilised, the modulation in response to air speed is clearly reduced. This supports previous findings from Heran^15^, which showed that the Johnston's organs are also responsible for regulating the honeybee's wingbeat amplitude in response to varying air speeds. Likewise, other studies have implicated the Johnston's organs in airflow sensing in butterflies^25^, flies^19^, dragonflies^26^ and locusts^27^ and have also suggested other mechanosensory roles (hearing^28^ and electric field perception^29^ in honeybees and inertial sensing in *Manduca*^12^). Indeed the Johnston's organs, and antennae more generally, are recognized to provide insects with a wide range of sensory measurements. However, in both kinds of antennal manipulation, we find that there is still some residual modulation in the abdominal response to airflow, and this activity can be predicted in both cases by our non-linear model if the insects are assumed to still have a mechanism to sense air speed. This suggests that other sensory receptors also contribute to the honeybee's perception of airflow, although these appear to have a reduced effect on the response compared to the information provided by the antennae. These additional sensory channels could be innervated hairs in various parts of the body, or wing load sensors, as found in many orders of insects^16^.

With the same information from the antenna being removed in both antennal manipulations, and likely the same alternate sensors providing air speed measurements, why does the response vary between bees with amputated antennas and waxed pedicels? The results of our non-linear model show that such results can be predicted, if honeybees make different adjustments to the way in which they process air speed information in the two cases. We speculate that this may be a mechanism of adapting to uncertain information. When a bee's antennae are amputated, it may be aware of this, and may then weight information from the correct, but possibly less sensitive, alternate sensors more strongly. When a bee's antennae are waxed it is less obvious they have been compromised, and the bee may not perform such reweighting, and even continue to use the erroneous air speed signals from its immobile Johnston's organ.

**Figure 6:**
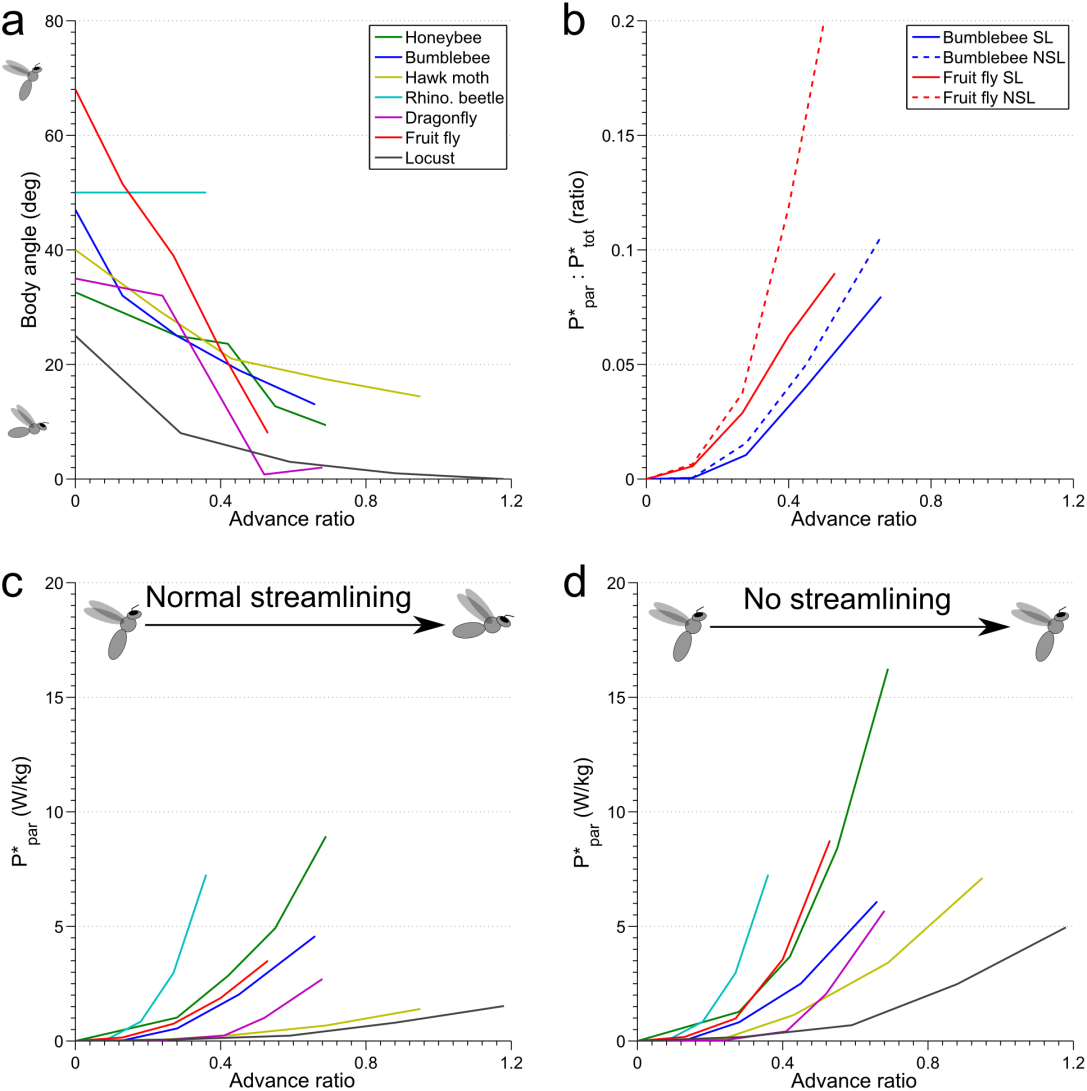
Streamlining reduces the power required for fast flight. (a) Body angle in free flight as a function of advance ratio for a variety of insect species ((a), (c) and (d)) use this legend). Note that this is a plot of body angle (the angle from the anterior tip of the head to the posterior point of the abdomen), rather than the abdomen pitch referred to elsewhere in this paper. By convention, this is plotted with positive 90° indicating a non-streamlined flight position, rather than negative 90° as we use for the abdomen pitch. (b) Ratio of parasitic power (per unit body mass) to total power (per unit body mass) required for flight at increasing advance ratios, when streamlined or not (SL: streamlined; NSL: not streamlined), for two species. (c) Parasitic power per unit body mass required for flight as a function of advance ratio when insects streamline as normal (using the body angles in (a) for each species). (d) As for (c), but the power is recalculated as if the insects had maintained the same body angle as for hovering (using the body angle at an advance ratio of zero for each species in (a)). The broken lines in (b) are calculated using the same approach. See Supp. 6 for details on this data, and references.

Adjustments in body angle were first observed in free flying, rather than tethered insects. In free flight, the majority of insects have a similar response to what we have observed in tethered honeybees. Figure 6a shows body angle plotted against the advance ratio (the ratio between forwards velocity and mean flapping velocity of the wingtip), and as flight speed, and hence the advance ratio increase, the pitch angle of the body is adjusted until it is almost horizontal. However, in free flight the thorax and head are *also* rotated forwards, which a tethered insect cannot do. The insects we consider in the comparison shown in Figure 6a vary in size by three orders of magnitude, as does the Reynolds number of their body over their flight speed ranges (Supp. 6) and yet most show a similar streamlining behavior.

In this paper we focus on active, behavioral adjustment of the body to reduce drag; however, insects also have some aspects of streamlining inherent to their morphology. This is the drag coefficient of an insect's body, which is usually compared between insects when the body is orientated with the long axis parallel to the air stream. This coefficient ranges from 0.25 in the case of *Manduca*^30^, which are very streamlined, to over 2.3 for small flies^31^, which are generally less streamlined. Yet, regardless of their inherent aerodynamic design, the majority of insect species make some attempt to streamline their flight. Does streamlining actually provide worthwhile benefits for insects? A comparison of Figure 6(c and d) reveals that, when the insect streamlines in the left hand plot, the mass specific parasitic power (the power used to overcome drag against its body), increases with advance ratio, but for all the insects this can more than double at the equivalent advance ratio if it does not streamline its body. This effect is most pronounced near the upper limits of the flight speed of each species.

Reduction in parasitic power is only meaningful if it represents a large contribution to the total power required for flight (a flying animal must also support its body weight which requires energy). The total power required for flight over a range of flight speeds has only been found, by simulation, for two species; *Drosophila virilis*^32^ and *Bombus terrestrius*^33^. The ratio of parasitic to total power for these two species is similar over their flight speed ranges, and whilst parasitic power represents a negligible portion of the total power at low advance ratios, it rises rapidly to near a tenth of the total power at higher flight speeds (Figure 6b). If each species were to not streamline, and maintained their hovering abdomen angle across all flight speeds, parasitic power costs at high air speeds would be even larger, approaching one fifth of the total power required by *Drosophila* (the increase for bumblebees is not so severe, as they start at a body angle 20° closer to horizontal). Although it has been suggested streamlining is of little benefit for small insects^34^, this statement appears to be only true when evolving an insect's body shape to be aerodynamically streamlined; regardless of their size or inherent drag coefficient, most of the insects considered here assume a streamlined body angle and benefit by reduced parasitic power, which might otherwise be a factor that limits their maximum flight speed.

An exception to the general trend for streamlining is the order *Coleoptera*, the beetles. The rhinoceros beetle we include in Figure 6 do not streamline, and consequently they are likely to experience a body drag that is much higher than in for other insects at comparable advance ratios. These insects also reach maximum flight speed at lower advance ratios than other large insects in our comparison (hawk moth and locust), suggesting that body drag may be a limiting factor on the upper limit for the flight speed that beetles can attain. In some cases, other beetles adopt a peculiar looking posture where the thorax is not re-oriented, but the head is thrust forward, and the abdomen is partially lifted up into a streamlined position^35^. Beetle flight is somewhat different from the other insects we have considered: whilst they have only a single pair of true wings, like Diptera, their front wings, or elytra, are hardened and more like armor than wings. Elytra no doubt provide desirable protective benefits, and in flight may generate close to a fifth of the lift required to keep the insect aloft (much like a fixed wing aircraft), and even beat in time with the rear wings, albeit at a smaller amplitude^36^. Thus whilst elytra may not have evolved to aid beetles in flight, their flight behaviors are probably optimized to make the most of their aerodynamic properties, possibly placing less emphasis on streamlining other body parts.

In theory, an insect should benefit from assuming a streamlined posture at any air speed above zero. Why, then, would a flying insect choose not to be streamlined at all flight velocities? When the abdomen is raised to a streamlined position it exerts the largest possible nose-up pitching moment on the insect. Whilst aerodynamic forces against the abdomen would act to raise the abdomen, our experiments, and calculations of the aerodynamic forces on free flying bumblebees^37^, show that this would be insufficient to sustain the abdomen in a streamlined position, even at high air speeds. Much as they must support the insect's weight with a vertical force when hovering, it seems that insects' wings must also generate a nose-down pitching moment to allow streamlining. Hoverflies hover with ‘streamlined' abdomen positions, and simulation studies have shown the power requirements for hover for these insects is only 10% more than when hovering with their abdomens lowered^38^. However, they do appear to have morphological adaptations that facilitate this; the relative distance from the center of mass to their wing base is approximately half that of many other insects, including other Diptera^39^, which would reduce the nose-up moment resulting from holding the abdomen up. Hovering with a raised abdomen may be more energetically taxing for other insects, in which case the choice to streamline could be a compromise.

We previously mentioned that an insect in free flight rotates its entire body forwards. This does not necessarily imply that the net force vector (i.e. the angle between thrust and lift) produced by an insect's wings rotates with its body as the insect can also adjust its stroke plane. In fact, whilst both *Drosophila*^40^ and bumblebees^41^ are observed to rotate their bodies by 40° or more over the range of their free flight speeds, simulation studies show that the direction of the net force vector produced by the wings only varies by around 10° for both insects^32^,^33^, suggesting that changing the angle of the net force vector via thoracic reorientation may not be required for fast flight (although tethered *Drosophila* flying in still air show an angle of the net force vector which is fixed to the thorax^42^). The streamlining response (relative to the insect's body) of tethered honeybees is invariant to the pitch of the tethered insect's thorax, as least for positive thorax pitch angles varying from 0° to 36°. Thus, a complementary purpose for thoracic reorientation may be to allow the abdomen to freely move into a streamlined position relative to the opposing airflow; the reduction in energy expenditure from streamlining may make sustained fast flight more achievable for many insects.

Whilst minimising energy expenditure (or maximizing flight speed given an energy budget), is a compelling reason to streamline the abdomen, there may be other considerations for insect flight. One reason for maintaining a low abdominal position may be to improve aerodynamic stability. Besides decreasing the nose-up pitching moment on the body, lowering the abdomen would increase the moment of inertia about the insect's roll axis, thus enhancing roll stability, and bring the insect's centre of mass forwards, which would enhance longitudinal stability^43^. This is not the only option available to insects; Combes and Dudley^44^ observed that tropical orchid bees extended their hind legs at high flight speeds to increase roll stability, at the cost of increased drag, but did not change their abdominal posture. Further, tethered *Drosophila*^45^ and *Manduca*^46^ have been observed to actively raise or lower their abdomen when shown a visual stimulus that indicates a pitch disturbance. This abdominal motion would both change the moment generated by the weight of the abdomen, and also create an inertial torque about the thorax-abdomen joint, both of which would act to adjust the pitch of the insect's thorax (provided the wings did not modify their generated pitching moment). In the case of *Manduca*, such a reaction has recently been calculated to be capable of providing pitch stability^46^.

Clearly, by changing their body posture, flying insects trade benefits from reducing energy expenditure with increased aerodynamic stability and control, and possibly other, as yet undiscovered factors. One potential application of such a control scheme would be for dynamically reconfigurable small aerial vehicles. For instance, the battery pack on such a robot could be placed and actuated in a similar way to an insect's abdomen, and this type of device has recently been shown to provide pitch stability for a quadrotor helicopter, based the control model discovered for *Manduca*^47^. However, on long distance, cruising flights it would be critically important to ensure that such an aircraft is streamlined, as body drag accounts for the majority of power requirements in human scale vehicles at higher velocities^48^.

## Methods

### Experimental animals

Adult forager honeybees (*Apis mellifera*, L) were used in all experiments. All insects were collected from a single hive maintained by the Queensland Brain Institute at The University of Queensland, Brisbane, Australia. Only foragers were collected, and were identified as those carrying pollen on their hind legs when returning to the hive.

### Tethering

Honeybees were cold anaesthetized in a refrigerator for 20–30 minutes, after which they were removed individually for tethering. Animals spent no more than one hour under anaesthesia. While the insect was anaesthetised, the base of an L-shaped metal rod was attached to the head and the thorax by a globule of dental adhesive (shade modification, SDI), which was cured using high intensity blue light (radii plus, SDI). Whilst this globule occulded the ocelli, this is unlikely to have affected the honeybee's behavior as the ocelli have a low spatial resolution^22^ that would not detect the movement of the grating used in this assay. Adhesion to the tether was facilitated by gently shaving the hair on the notum using a scalpel.

Antennal manipulations were performed after tethering whilst the honeybee was still anaesthetised. In the case of amputation, the antennae were cut close to the base using a pair of surgical scissors (Figure 3d). Waxing of the antenna was performed using dental wax applied with a hand cauteriser (Change-A-Tip Deluxe Cautery Kit, Bovie Medical Corporation). The antenna was positioned such that the two segments, the flagellum and the scape were approximately at right angles (their normal orientation), and the hot wax was touched lightly to the joint. The wax wicked into the pedicle joint, and also across the flagellum; the success of immobilization was tested by gently attempting to bend the antenna with a pair of forceps (Figure 3c and e). The integrity of the waxing was tested both before and at the conclusion of the experiments, and honeybees were rarely found to have removed the wax (those that had were not included in the analysis). Honeybees undergoing this preparation still exhibited the proboscis extension reflex in response to sugar water touched on their antenna, suggesting that the antennal nerve was still intact. In experiments with manipulated antennae, honeybees were generally more reluctant to fly, and those with waxed Johnston's organs often tried to groom the wax off.

Tethered honeybees were housed in a Styrofoam box placed on a heater, which maintained the temperature at 28–30°C. A beaker of water, placed inside the box, provided an environment with the appropriate humidity. Insects typically recovered from anaesthesia after several minutes in the humid box, and were then fed with several drops of 1 mol/L sucrose solution, and had at least 30 minutes to recover before their first flight.

### Visual stimulus display and generation

Images were displayed on four 22″ monitors (2209 WAf, Dell) arranged in a diamond shaped arena to provide a near panoramic virtual environment. The tethered insect was positioned in the center of the arena by attaching the tether to a clip held in the arena by an articulated arm (MA61003, Noga), with the honeybee's head facing a corner of the arena. The LCD monitors were driven by a computer (Intel i7 CPU (4 core @2.67 GHz), 2.5 GB RAM, Windows XP SP 3), with two dual head NVIDIA GeForce GTX 260 video cards. The monitors were configured to use 1680 × 1050 pixel resolution at a 60 Hz update rate. The dimensions of the monitor screens were 475 × 300 mm, and the diamond arrangement covered approximately 61° of the tethered honeybee's vertical visual field at the closest point and 45° at the corners (both full angles). The black plastic frames of the monitors prevented a full 360° coverage of the insect's azimuthal visual field, leaving four gaps, each approximately 3° wide, in the front, the rear and the two sides.

As shown in Figure 1, the computer monitors provide panoramic visual stimulation in the horizontal plane, with non-stimulated areas in the dorsal and ventral view fields. Whilst not entirely true to the world an insect would observe in natural flight, the apparatus was designed to replicate the stimulus used in Luu et al.^8^ as closely as possible (the monitors are 2″ smaller), to enable a direct comparison of the role of air speed in modulating the visually mediated streamlining response discovered in that study. The response to visual motion alone (at 0 m/s, Figure 2a) saturates at approximately 10 deg/s lower than that observed in the previous study, but is otherwise qualitatively similar, suggesting that the smaller monitors, and areas of the monitor occluded by the fan for air flow generation, described in the following section, may have slightly reduced the strength of the visual input in stimulating the response. The visually evoked component of the streamlining response has previously been found to be the result of a non-linear spatial summation of optic flow seen across all angles of elevation^8^ (within ± 23° of the insects transverse plane), and so including stimulus in the dorsal and ventral view fields may not have any substantial effect, as the response is already close to saturation.

A custom written C++ program was used to generate the visual display. This display was similar to that used by Luu et al.^8^, where motion was simulated along an apparently infinitely long tunnel of user-selectable width, displaying red and white sinusoidal gratings on the inside walls, with a spatial frequency of 0.014 cycles/deg. At the maximum optic flow used (600 deg/s), the 60 Hz update rate resulted in images ‘stepping' at 0.14 cycles/frame. For a honeybee flying along a virtual tunnel, the maximum image velocity as experienced by the eyes occurs in the lateral viewing direction, i.e. in a viewing direction at 90° to the direction of flight. The values of optic flow shown in the graphs correspond to the values pertaining to this viewing direction.

### Air flow generation

Air flow, or simulated wind, was generated by two fans (TurboFan 12 VDC 40 × 28 MM 20000 RPM, NMB Technologies Corp.) connected in series, which blew wind through a square shaped wind tunnel that incorporated a honeycombed cross section to reduce the turbulence of the flow. The end of the tunnel was approximately 140 mm in front of the insect, and subtended 22° (full angle, vertically and horizontally) of the honeybee's frontal field of view. Whilst this is a sizable portion of the insect's visual field, we do not believe that its presence would have affected the visually-driven component of the streamlining response, as the frontal visual field has been shown to have minimal influence on insect's streamlining response and flight speed regulation^8^,^49^.

The speed of the fan was controlled by a pulse width modulated (PWM) signal, and the air speed was measured by an anemometer. The PWM signal was generated by a USB data acquisition (DAQ) module (U3-HV, Labjack) which was controlled in real time by a program running on the PC. The DAQ module also acquired data from the anemometer positioned behind the insect (EE-65VB, E + E Elektronik Ges.m.b.H). The PWM signal versus air speed relationship was calibrated by placing the anemometer at the insect's usual flight position and measuring the air speed resulting from 5% PWM increments. The PWM required for a desired air speed was then found by interpolating between the calibration points. Turbulence, measured as the standard deviation of the air speed over a time interval of thirty seconds, increased in absolute value as the air speed increased, whilst the ratio of the standard deviation to the mean air velocity remained at approximately 5% for velocities up to 5 m/s. The airspeed was held constant during any given experimental trial (in which the optic flow was varied systematically).

Initial experiments showed that honeybees exhibited a hysteresis-like effect when exposed to different air speeds. Further investigations revealed that the order of presentation of air speed was significantly affected the honeybees response, whereas there was no variation dependent on the order optic flow was presented (Supp. 4). To avoid this confound, individual honeybees were only stimulated with a single constant air speed during their test in the arena (notwithstanding the transient ramps up and down at the start and end of the test protocol). For any given trial, the air speed was assigned randomly; hence measurements of data at different air speeds are independent.

### Data acquisition and image analysis

A camera (FireFly, Point Grey) filmed the side view of the bee (perpendicular to the simulated direction of flight, at 30 fps) against a piece of white paper (8 × 8 cm, placed at the opposite corner of the stimulus arena from the camera). Video frames were recorded with a time stamp linked to the stimulus. The orientation of the honeybee's abdomen was measured in real time for each frame using a custom written C++ program written in-house. The program tracked the axis of the insect's abdomen, and found its angle relative to the user-defined orientation of the thorax. This was defined as the ‘abdomen angle' or ‘response'. Examples of abdomen tracking are shown in Figure 1(d and e). The response was defined to be positive or negative, according to whether the abdomen was elevated or depressed relative to the axis of the thorax. The abdominal angles reported throughout this study are of the honeybee's steady-state response to each of the six stimulus speeds. The steady-state response was calculated as the mean abdominal angle measured during the last 5 s of each 10 s epoch of stimulus speed. Typically, the abdominal response reached a steady state within 1–3 s following the presentation of each new stimulus speed.

### Statistical analysis

All statistical analyses were performed using IBM SPSS Statistics V20. Main effects were tested using ANOVA. Prior to conducting ANOVA, we conducted tests of normality, homoscedasticity and sphericity. The vast majority of data was normally distributed, but failed tests of homoscedasticity and sphericity. Sphericity was corrected using the Greenhouse-Geisser correction, and we discuss the effect of heteroscedasticity on our analysis in Supp. 7, as well as providing full details of all statistical tests. Flight data from bees were included for analysis only if the bees flew continuously through the entire optic flow ramp. Four to six trials were conducted per honeybee to reduce inter-animal variability, and data points from multiple flights of an individual bee were averaged.

### Flight protocol

For each flight trial, a tethered honeybee was removed from the humid box and placed in the center of the arena and allowed to hold a small piece of waxed paper. As soon as the visual stimulus and air flow were started, the paper was removed from the honeybee's grasp and the tarsal reflex initiated flight. At the end of each flight the honeybee was returned to the humid box and offered several drops of 1 mol/L sucrose solution. Animals were rested for at least 20 minutes between consecutive trials. We used a stimulus protocol that simulated flight at a progressively increasing flight speed, namely, 100, 200, 300, 400, 500 and 600 deg/s. Each epoch of stimulus speed was 10 s in duration, thus each trial lasted 60 s. Air flow was started prior to the start of visual motion, and was maintained at a constant level through each trial.

## Author Contributions

G.J.T. completed experiments, analysis, modeling and wrote the manuscript. T.L. assisted in experimental design and analysis. D.B. assisted in experimental design and edited the manuscript. M.V.S. assisted in all aspects of the work and edited the manuscript.

## Supplementary Material

Supplementary Information Supplementary material
