# Supplementary material for: Vision and air flow combine to streamline flying honeybees
Source: Sci Rep. 2013 Sep 10;3:2614. doi: 10.1038/srep02614 (PMC3767942; doi:10.1038/srep02614)
Supplement: Supplementary Information — Supplementary material [file srep02614-s1.pdf]

Supplementary material

Vision and air flow combine to streamline flying honeybees

Gavin J. Taylor[1,2]*, Tien Luu[1], David Ball[3,4], Mandyam V. Srinivasan[1,2,4]

[1]Queensland Brain Institute, The University of Queensland. Brisbane, QLD, 4072, Australia, [2]ARC Centre of Excellence in Vision Science, The Australian National University. Canberra, ACT, 0200, Australia, [3]School of Electrical Engineering and Computer Science, Queensland University of Technology. Brisbane, QLD, 4000, Australia [4]School of Information Technology and Electrical Engineering, The University of Queensland

*Author for correspondence (gavin.taylor@uqconnect.edu.au)

Contents:

1. **Response to a tail wind**
2. **Passive lifting by air flow**
3. **Invariance of the response to thorax orientation**
4. **The influence of stimulus presentation order**
    a. **Air speed**
    b. **Optic flow**
5. **Models of interaction between air speed and optic flow**
    a. **Details of models**
    b. **Antennal manipulation comparison**
6. **Comparison of streamlining benefits across insect orders**
7. **Statistical tests**
    a. **Test of effects of air speed and optic flow**
    b. **Test of effects of air speed and optic flow on antenna amputated bees**
    c. **Test of effects of air speed and optic flow on antenna waxed bees**
    d. **Test of effect of antennal manipulation**
    e. **Test of effect of wind on decapitated bees**
    f. **Test of effects of negative air speed (tail wind) and optic flow**
    g. **Test of effect of optic flow presentation order**
    h. **Test of effect of air speed presentation order**
    i. **Test of effect of air speed and optic flow when the thorax is pitched up**
    j. **Test of effect of air speed and optic flow when the thorax is pitched down**
8. **Supplementary references**

1. Response to a tail wind

We examined how tethered honeybees respond to a tail wind by positioning the fan behind a tethered honeybee in the arena, and conducted experiments similar to those in the antennal manipulation case (at air speeds of -0, -0.5, -1.5 and -3 m/s, corresponding to the points of interest in the positive air speed response). At these negative air speeds, honeybees still displayed their characteristic streamlining response to the visual stimulus (Figure S1). The responses at -1.5 and -3 m/s were close to the baseline ‘no wind’ condition, whilst the response at 0.5 m/s was slightly elevated. ANOVA testing revealed that, with tail winds, the streamlining response depends weakly on airspeed ($F_{3,32}$=3.10, p=0.041), with significant differences being observed between the response at -0.5 and -1.5 m/s (Section 7.f).

Whilst the observed response to a tail wind is clearly different from a honeybee’s response to a head wind, it is not clear if this is because the honeybee is unable to sense the air speed (because its antenna are occluded by its body), or senses the air flow, either with its antenna or with other sensory organs, and chooses not to respond. However, the results indicate that the streamlining response is functionally asymmetrical, with the response at positive air speeds differing from the response at negative air speeds, much as the streamlining response to optic flow[1].

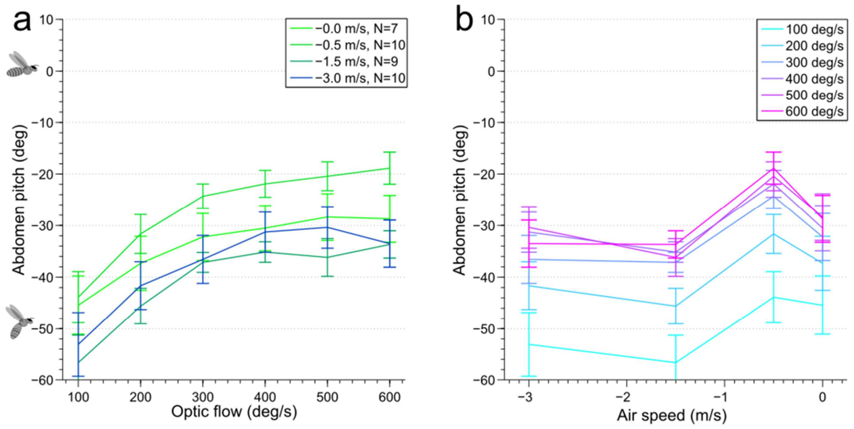

Figure S1: Response to a tailwind: Plotted as a function of optic flow with air speed as a parameter (a), and as a function of air speed with optic flow as a parameter (b). (a) Box shows number of bees tested at each air speed. Error bars show ± s.e.m.

2. Passive lifting by air flow

It is possible that airflow from the fan acted to lift the abdomen passively, by causing a pitching moment about the thorax-abdomen joint, generated by the aerodynamic force against the abdomen, to pivot it into a streamlined position. For this to occur, the aerodynamic pitching moment would have to exceed the opposing moment caused by the weight of the abdomen itself. Since aerodynamic forces increase as the square of the wind velocity, high air speeds could exert an appreciable force against the insect’s abdomen.

The effect of drag force on passive abdomen lift was investigated by using honeybee bodies that would experience the same drag forces as living bees. Honeybees were collected from the same hive as the insects used in the other experiments. They were tethered as usual, and then euthanized by amputation of the head capsule before they had recovered from the cold anaesthesia. The thorax and abdomen were immediately tested using air speeds of 0, 1, 2, 3 and 4 m/s. Prior to testing, the abdomen was manually positioned at approximately -90°, which should have exposed it to the maximal possible drag force for a given air speed. Reflexive motions of the legs were observed for up to an hour after decapitation, indicating that rigor mortis had not set in.

The results indicate that air flow over the abdomen of deceased bees causes a small increase (several degrees) in the abdomen posture (Figure S2). In addition to aerodynamic forces, the thoracic ganglia could have caused abdominal motion either randomly, or based on information from air speed sensing organs on the body that projected directly to it, and these motions would have been combined with those caused by external forces. However, ANOVA (Section 7.e) showed no significant effect of air flow ($F_{4,34}$=0.83, p=0.517) on abdomen posture, and the range of the response exhibited by live flying honeybees far exceeded that of the decapitated insects, even at the lowest speed of optic flow (Figure S2). The aerodynamic forces clearly play a minor role in lifting the abdomen.

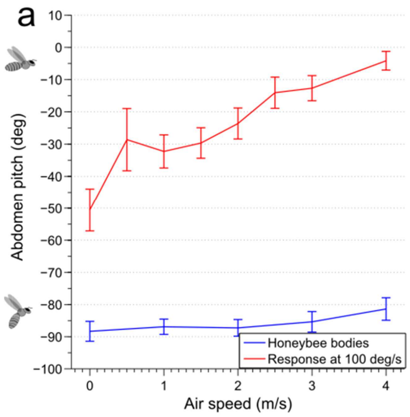

Figure S2: Passive forces acting on the abdomen. (a) Effect of air speed on the abdomen pitch of deceased honeybee bodies, compared to the response of live tethered honeybees experiencing an optic flow of 100 deg/s (the lowest optic flow condition). 7 deceased bees were tested. The sample sizes used for the live bees at each air speed are shown in the legend for Figure 2a (main text). Error bars show ± s.e.m.

3. Invariance of the streamlining response to thorax orientation

During natural flight honeybees reorient their entire body as their flight speed increases[2] (as do many other insects, see Section 6). In this situation, the head, thorax and abdomen are rotated forwards relative to the flight path, but the thorax – abdomen angle (which we describe as abdomen pitch in this study) remains relatively constant. Such a rotation would change the angle of the insect's head, and the sensory structures on it, relative to the oncoming optic flow and air flow, which in this study are directed horizontally. To test if the angle of the thorax affected the abdominal pitch response to optic flow and air speed, we conducted experiments where bees were tethered with the thorax pitched up, simulating slow or hovering flight, and the thorax

pitched down, a position that is not assumed during normal flight. These results were compared to those obtained for the usual experimental paradigm where bees were tethered with their thorax at 0°, simulating fast flight. These angles are of the thorax relative to the horizontal, and are analogs of the body angle measured for free flying insects (although in free flight the abdomen is usually held in line with the thorax). The effects of air speeds that would elicit a low, intermediate, and strong streamlining response were also tested, these air speeds being 0, 1.5 and 3 m/s respectively.

The thorax angle of bees could only be set by changing the angle they were glued relative to the metal tether; it was not possible to adjust this during or between experiments. The insects often refused to participate in experiments if they were anesthetized a second time, and because of this, separate bees were tested for three thorax orientations. The average thorax angle of bees with their thorax pitched down was -33 ± 4.6^{o}, and the average with their thorax pitch up was 36 ± 5.3^{o} (± s.d.). In addition, we used data collected with the thorax pitch horizontal (2 ± 4.2^{o}) from the standard experiments described in the main paper. Notably, bees with their thorax pitched down were very reluctant to fly (even more so than when antennal manipulations were performed); indeed, the flight attitude appears very unnatural (Figure S3). In these experiments the bees were only tested at a single air speed because there was an effect of presentation order on the response to air speed, as described in the following section.

Bees with their thorax pitched up show a similar response (Figure S3a and d) to optic flow and air speed as do normal bees (Figure S3b and e). Increasing strengths of optic flow and air speed both act to increase the response, and they appear to interact via a non-linear, saturating function. Both main effects (air speed and optic flow), and their interaction, are significant (Section 7.i). Furthermore, there is no statistical difference between bees in this condition and bees with their thorax tethered horizontally, neither as a main effect of tethering angle, nor as an interaction with any other factors (Section 7.i). Hence, it appears that at the upper and lower limits of thorax angles that a bee might assume during natural flight (at the three wind speeds tested), the streamlining response persists, regardless of thorax angle.

Conversely, honeybees with their thorax pitched down show a qualitatively different behaviour (Figure S3c and f); noticeably optic flow no longer acts to steadily increase the abdomen pitch to a plateau point. In fact, for all three air speeds, the response relative to optic flow reaches a peak value before the maximum optic flow rate tested, after which it begins to decrease. Furthermore, the responses at 0 and 1.5 m/s air speed overlap substantially, suggesting that the response to air speed is also modified by pitching the thorax down. ANOVA shows there is no longer a significant main effect of optic flow, although it continues to interact with air speed, which is itself significant (Section 7.j).

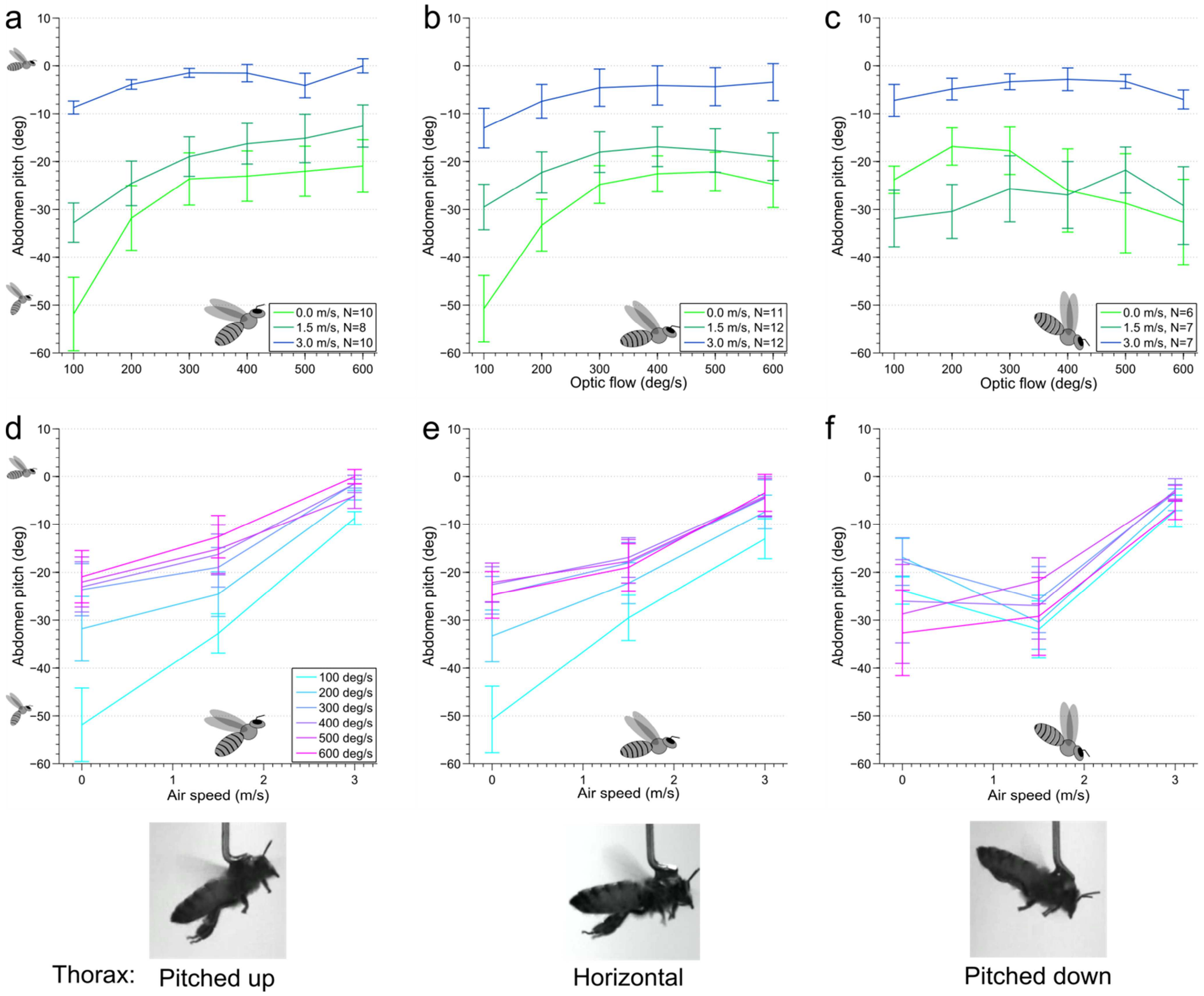

Figure S3: The honeybee's abdomen response when tethering angle of the thorax is varied. The response is plotted as a function of optic flow with air speed as a parameter (a, b, c), and as a function of air speed with optic flow as a parameter (c, d, e). (a, d) represent data with their thorax pitched up, (b,d) from bees with a horizontal thorax (re-plotted from Figure 2 (main text)), (c, f) from bees with their thorax pitched down. The legends in (a, b, c) show the sample size of bees tested at each airspeed, for a particular thorax pitch. The legend in (d) is also used for (e, f). The image at the bottom of the figure show representative images of bees at different thorax tethering angles, flying in a 3 m/s air stream. Error bars show ± s.e.m.

4. The influence of stimulus presentation order

Initial experiments showed that honeybees exhibited a hysteresis-like effect when exposed to different air speeds. Qualitatively, it appeared that when the insects were initially exposed to a high air speed, they were reluctant to fly when the speed was lowered or removed, perhaps finding it to be unnatural. This effect was observed to occur regardless of whether the lower air speed was presented to a bee during the same flight trial as the higher air speed, or during a later trial. To test this apparent effect of presentation order, or hysteresis, we trialed honeybees with air speed levels represented by a stepped triangle function of increasing and then

decreasing air speeds, at several optic flows. Whilst no dependence of the response to optic flow on presentation order had been observed, we also tested separate bees with a similar function of increasing then decreasing optic flows, at several air speeds.

a. Air speed

Honeybees were exposed to a stimulus pattern of progressively increasing series of air speeds, from 0 - 5 m/s (in 1 m/s increments of 10s duration), followed by a decreasing series of air speeds that mirrored the initial increase. Honeybees were tested at optic flow levels that would elicit low, intermediate and strong streamlining responses, these being 100, 300 and 500 deg/s. This was similar to the standard protocol of presenting a progressively increasing series of optic flow levels, except here optic flow was held constant during the flight whilst air speed was systematically varied. Bees were exposed to all optic flow levels twice in random order, and their responses averaged.

When exposed to this triangular function of air speed levels, honeybees typically maintained their abdomens at a higher level during the decreasing portion of the response function, regardless of optic flow level (Figure S4). The sole exception is at the end of the ramp when there is no wind, or at 3m/s air speed, at which point honeybees dropped their abdomens to lower positions than at the beginning. Generally, this phenomenon appears to approximate a classic hysteresis function. The effects of optic flow, air speed and their interaction in these trials were significant, and agreed with other results in the manuscript (Section 7.h). The effect of air speed presentation order was not itself significant as a main effect ($F_{1,9}$=0.76, p=0.407), but showed a significant interaction with air speed ($F_{1.6,14.4}$=11.44, p=0.002), and also a three factor interaction with air speed and optic flow ($F_{2.0,17.7}$=5.41, p=0.015). Thus, it appears the order in which a tethered bee experiences air speeds modifies its response to the following air speeds, and further how this response interacts with the response to optic flow.

It is unclear what causes presentation order to affect the observed abdomen pitch in this manner. The response itself does not display hysteresis when bees are exposed to a triangular function of optic flow (see the following section), and a similar effect was initially observed when air speed was changed between trials, suggesting that the response does not arise from short term habituation of the mechanoreceptors. We speculate that tethered honeybees rapidly become accustomed to higher air speeds in the flight arena, and are reluctant to assume a posture associated with low flight speed. They may even modulate their flight forces during the decreasing section of the triangular function, in an attempt to accelerate to faster flight speeds. Regardless of the cause, such hysteresis may be a consideration in the design of experiments investigating flight control of insects in response to varying air speeds, and understanding the underlying mechanosensory cues.

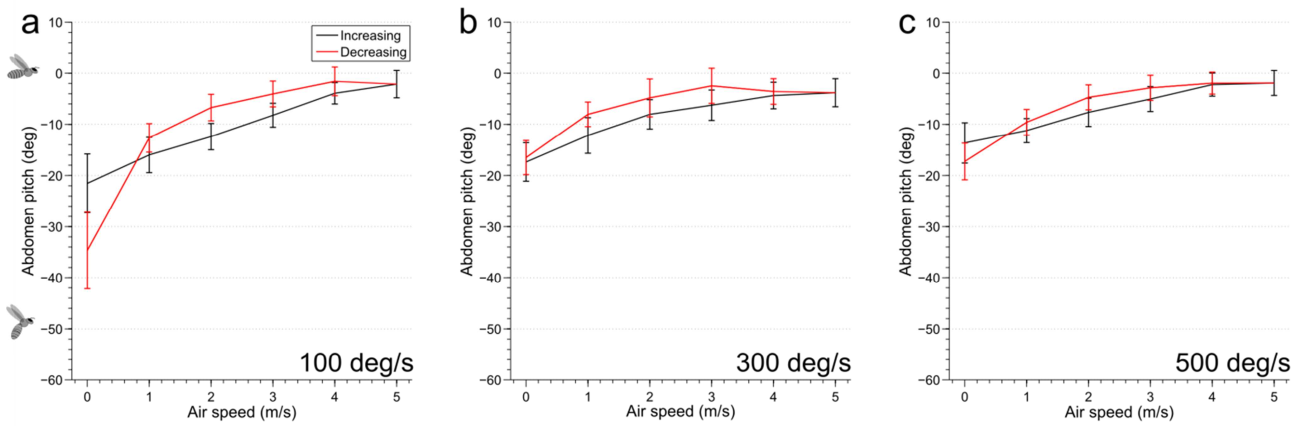

Figure S4: Air speed presentation order affects the streamlining response. Response to a triangular air speed function at various optic flow levels: (a) 100 deg/s, (b) 300 deg/s and (c) 500 deg/s. Presentation order is indicated as increasing (black line), followed by decreasing (red line) air speed. 10 bees participated in all conditions. Error bars show ± s.e.m

b. Optic flow

Honeybees were exposed to a standard stimulus of progressively increasing optic flows, going from 100 – 600 deg/s (in 100 deg/s increments of 10s duration), followed by a decreasing series of optic flows that mirrored the initial increase. As presentation order was already suspected to affect the response, separate honeybees were tested without air flow and with airflow at intermediate and high speeds with respect to their effect on the streamlining response (Figure S5). These air speeds were 0, 1.5 and 3 m/s respectively.

Whilst there is some variation of the response with the increasing and decreasing side of the triangular optic flow function, this variation appears to be inconsistent and varies qualitatively between air speeds. Again, optic flow and air speed show a substantial effect on abdomen position, and indeed these are the only significant effects detected (Section 7.g). The presentation order of optic flow has no significant effect on streamlining per se, nor does it show any significant interaction with other parameters. Thus, we conclude that the order of presentation of optic flow does not need to be controlled when designing experiments to examine its effects.

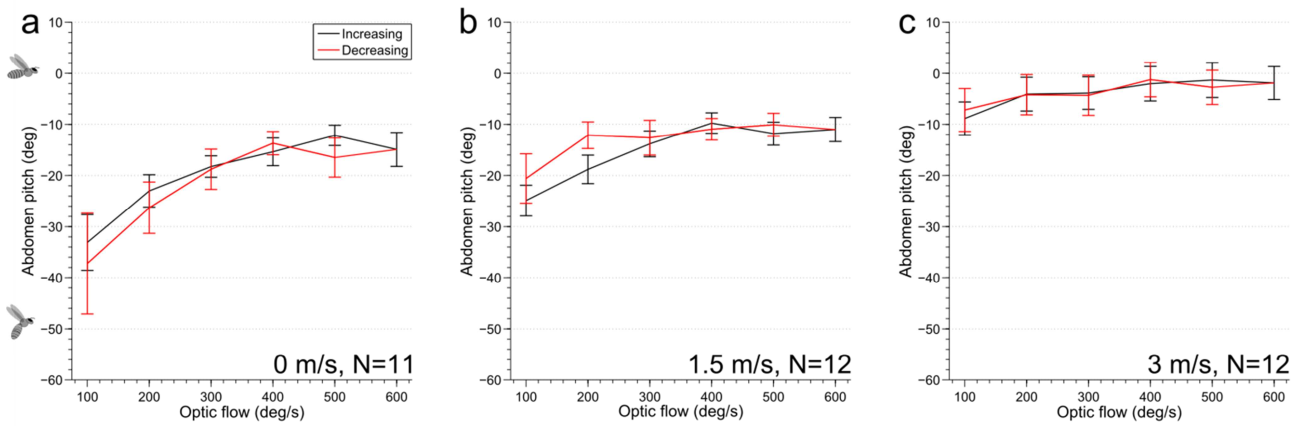

Figure S5: Optic flow presentation order does not affect the streamlining response. Response to a triangular optic flow function at various air speed levels: (a) 0 m/s, (b) 1.5 m/s, (c) 3 m/s. Presentation order is indicated as increasing (black line), followed by decreasing (red line) optic flow. The number of bees tested for each air speed are indicated in the bottom right corner of each plot. Error bars show ± s.e.m

5. Models of interaction between air speed and optic flow (Figure 4 (main text))

5.a Details of models

All of the saturating functions are characterized by a 4 parameter variable slope sigmoidal function, represented by the equation:

$$Y(x) = p(1) + \frac{p(2)}{1 + e^{(p(3)-x)\times p(4)}}$$

Here x represents input and Y(x) represents the output at a particular value of x. p(1) represents the minimum value of the function (or vertical offset), p(2) the response range of the function, p(3) the x value where the function is halfway between its minimum and maximum values (or, equivalently, the horizontal offset), and p(4) a measure of the slope of the curve at this point.

The best-fitting values of these parameters for the first four models were found using the least squares fitting tool 'nlinfit' in Matlab R2009b. The parameters of the non-linear combination model were found using a genetic algorithm optimisation approach, the function 'nsga2' from the Mathworks file exchange[3], where an artificial population size of 100 was used for 100 generations to optimise the 10 free variables using a least squares fit.

The values of these parameters for each of the saturating functions are given below (in all cases Figure 4 refers to the main text):

Figure 4a. Optic flow saturation function. (The minimum of this function, p(1), was constrained to -90°)

Sat.$_{OF}$: $AbP(OF) = -90 + \frac{66.9}{1+e^{(74.3-OF)\times 0.01}}$

Figure 4b. Air speed saturation function. (The minimum of this function, p(1), was constrained to -90°)

S

at.$_{AS}$: $AbP(AS) = -90 + \frac{90.1}{1+e^{(0.34-AS)\times 0.7}}$

Figure 4c. Abdomen pitch as a linear summation of the saturating response to optic flow and air speed.

AbP(OF,AS) = Sat.$_{OF}$ + Sat.$_{AS}$ + 90

$$AbP(OF, AS) = -90 + \frac{66.9}{1+e^{(74.3-OF)\times 0.01}} + \frac{90.1}{1+e^{(0.34-AS)\times 0.7}}$$

Figure 4d. Abdomen pitch as a weighted linear summation of the saturating response to optic flow and air speed.

AbP(OF,AS) = G_1×Sat.$_{OF}$ + G_2×Sat.$_{AS}$ + 90, Where G_1 = 0.45 and G_2 = 0.74

$$AbP(OF, AS) = -90 + \frac{30.1}{1+e^{(74.3-OF)\times 0.01}} + \frac{66.7}{1+e^{(0.34-AS)\times 0.7}}$$

Figure 4e. Abdomen pitch as a nonlinear combination of saturating responses to optic flow and air speed. Note that there appeared to be considerable 'play' in the optimiser's results, as the majority of variables could vary by some amount whilst still producing similar model results, indicating that the effect of various interacting variables tended to produce multiple solutions.

AbP(OF,AS) = Sat.$_{AS1}$+ Sat.$_{AS2}$× Sat.$_{OF}$,

where the air speed saturation function is:

S

at.$_{AS1}$: $-7.1 + \frac{-90.2}{1+e^{(0.8-AS)\times -1.1}}$,

the air speed saturating function that modulates optic flow gain is:

S

at.$_{AS2}$: $9.2 + \frac{40.8}{1+e^{(1.1-AS)\times -4.2}}$,

and the optic flow saturating function is:

S

at.$_{OF}$: $\frac{1}{1+e^{(124-OF)\times 0.01}}$

5.b Antennal manipulation comparison

The model using a non-linear combination of saturating response was tested against the results found for the two antenna manipulation cases. As these abdomen positions for antenna manipulated bees were different from those observed in normal bees, the model would obviously have to be adjusted to fit this data. It is of interest to find if an entirely new model would be required, or if the same method of combining air speed and optic flow could be used with adjusted parameters, and if that was the case, which parameters should be adjusted.

We refit the following model parameters, whilst keeping the other parameters at the same level as found for normal bees (the data was refit for antenna amputated and pedicel waxed bees separately):

- All parameters: (SatAS1 – p(1), p(2), p(3), p(4); SatAS2 – p(1), p(2), p(3), p(4); SatOF –p(3), p(4))
- Both saturating responses to air speed (SatAS1 – p(1), p(2), p(3), p(4); SatAS2 – p(1), p(2), p(3), p(4))
- Saturating response to optic flow (SatOF –p(3), p(4))
- Saturating response to air speed (SatAS1 – p(1), p(2), p(3), p(4))
- Saturating response to air speed that modulates optic flow gain (SatAS2 – p(1), p(2), p(3), p(4))

The results of refitting these parameters are shown in Table S1. Qualitative comparison between the various models and the corresponding data is done using plots of the models' output, as shown in Figure 4 (main text) and Figure S6. Similar to the model fit for data from non-manipulated bees, there appeared to be considerable 'play' in the parameters fit by the optimiser, further highlighting the interaction of variables in determining the best fit.

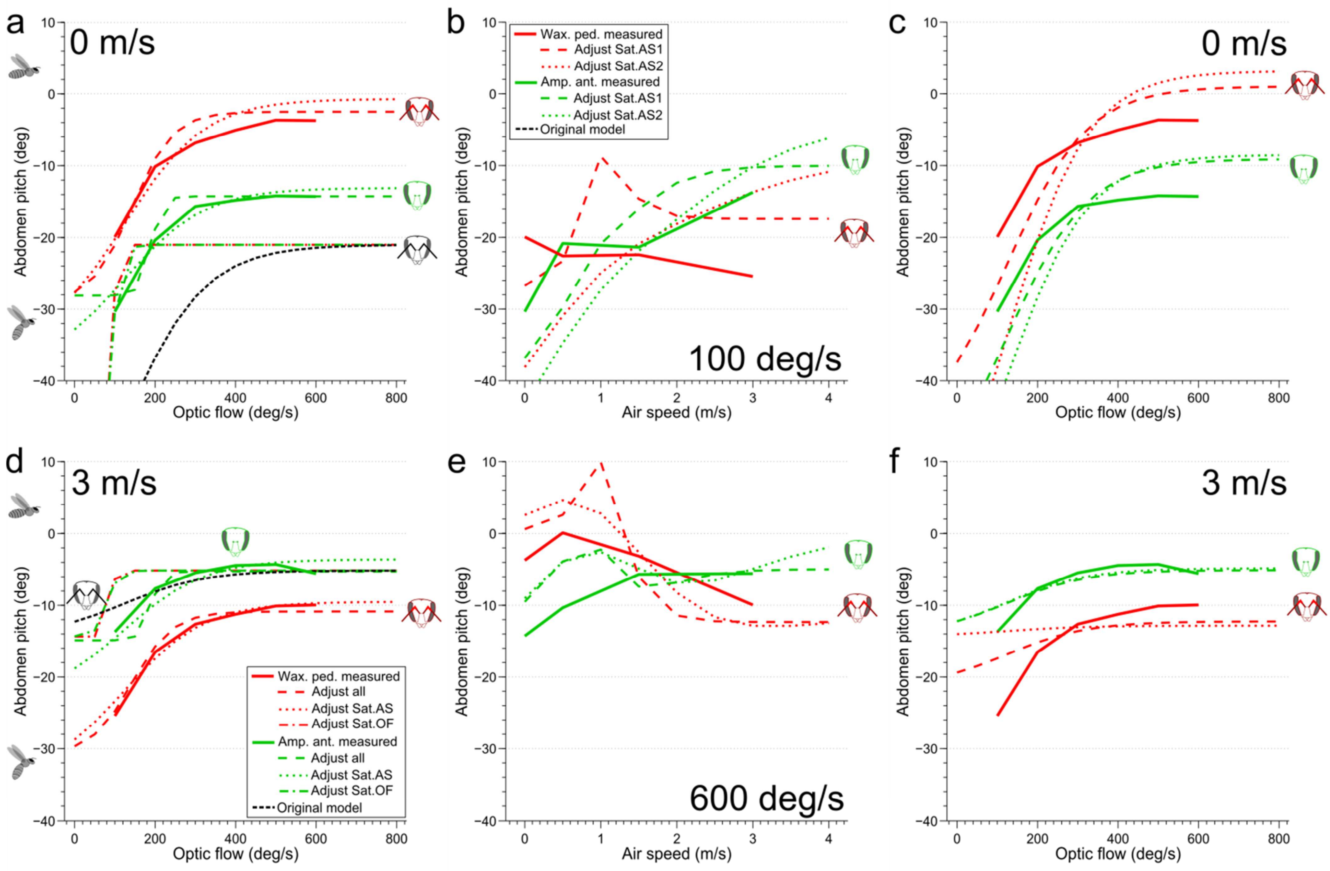

Figure S6: Adaptability of a non-linear combination of saturating response model to antennal manipulations. (a, d) Comparisons of the model fits for the manipulated antennae shown in Figure 4 (main text) at the low and high air speed boundaries of the tested response surface (plots of the low and high optic flow boundaries are shown in Figure 4 (main text)): (a) Response vs. air speed at 0 m/s air speed, (d) Response vs. air speed at 3 m/s air speed. (b, c, e, f) comparison of other model fits (the legend in this plot is used for (a, d)) for manipulated antenna, at the boundaries of the measured response surface. Different combinations of parameters are refit (for each manipulation case separately) whilst some are held constant with those found for non-manipulated bees (see text and Table S1 for further details): (b) Response vs. air speed at 100 deg/s optic flow (the legend in this plot is used for (b, c, e, f)), (c) Response vs. optic flow at 0 m/s air speed, (e) Response vs. air speed at 600 deg/s optic flow, (f) Response vs. optic flow at 3 m/s air speed.

Table S1: Comparison of model results for the various cases of antennal manipulation, and various constraints. For brevity the antennal manipulation cases are indicated in the left most column based on the following colour scheme: black, normal bees; green, antenna amputated; red, pedicel waxed. Model parameters that were fixed (at the value found for model fit for normal bees), are italicized, whereas those that were allowed to vary are shown in bold.

Model	Sat. AS1				Sat. AS2				Sat. OF	
	Vertical offset p(1)	Vertical range p(2)	Horizontal offset p(3)	Slope p(4)	Vertical offset p(1)	Vertical range p(2)	Horizontal offset p(3)	Slope p(4)	Horizontal offset p(3)	Slope p(4)
**Original model** (all points)	**-7.1**	**-90.2**	**0.8**	**-1.1**	**9.2**	**40.8**	**1.1**	**-4.2**	**124**	**0.01**
**Original model** (reduced set)	*-7.1*	*-90.2*	*0.8*	*-1.1*	*9.2*	*40.8*	*1.1*	*-4.2*	*124*	*0.01*
**Original model**	*-7.1*	*-90.2*	*0.8*	*-1.1*	*9.2*	*40.8*	*1.1*	*-4.2*	*124*	*0.01*
**Original model**	*-7.1*	*-90.2*	*0.8*	*-1.1*	*9.2*	*40.8*	*1.1*	*-4.2*	*124*	*0.01*
**Adjust all terms**	**-14.3**	**-25.3**	**0.1**	**-1.3**	**4.8**	**17.0**	**0.3**	**-0.4**	**190**	**0.07**
**Adjust all terms**	**-38.9**	**16.7**	**0.8**	**-0.1**	**20.0**	**6.7**	**1.8**	**-7.3**	**142**	**0.02**
**Adjust SatAS (both)**	**-23.1**	**-21.9**	**0.45**	**-1.9**	**19.6**	**5.8**	**1.0**	-20.9	*124*	*0.01*
**Adjust SatAS (both)**	**-30.6**	**-9.7**	**0.4**	**-0.2**	**9.2**	**26.1**	**3.3**	-1.3	*124*	*0.01*
**Adjust SatOF**	*-7.1*	*-90.2*	*0.8*	*-1.1*	*9.2*	*40.8*	*1.1*	*-4.2*	**81**	**0.07**
**Adjust SatOF**	*-7.1*	*-90.2*	*0.8*	*-1.1*	*9.2*	*40.8*	*1.1*	*-4.2*	**92**	**0.23**
**Adjust SatAS1**	**-14.1**	**-48.8**	**0.9**	**-2.6**	*9.2*	*40.8*	*1.1*	*-4.2*	*124*	*0.01*
**Adjust SatAS1**	**-21.5**	**-27.2**	**0.7**	**-8.0**	*9.2*	*40.8*	*1.1*	*-4.2*	*124*	*0.01*
**Adjust SatAS2**	*-7.1*	*-90.2*	*0.8*	*-1.1*	**7.4**	**59.4**	**1.3**	**-1.9**	*124*	*0.01*
**Adjust SatAS2**	*-7.1*	*-90.2*	*0.8*	*-1.1*	**-4.1**	**87.7**	**1.3**	**-1.5**	*124*	*0.01*

6. Comparison of streamlining benefits across insect orders (Figure 6 (main text))

Figure 6 (main text) is the result of a meta-analysis of the drag forces incurred during flights at a range of forward air speeds for a collection of seven insect species, across six orders. These are:

1. Hymenoptera
    a. *Apis mellifera* (honeybee (female worker))
    b. *Bombus terrestris* (bumblebee (female worker))
2. Lepidoptera - *Manduca sexta* (hawk moth (male))
3. Coleoptera - *Trypoxylus dichotomus* (rhinoceros beetle (male))
4. Odonata - *Sympetrum sanguineum* (dragon fly)
5. Diptera - *Drosophila viralis* (fruit fly)
6. Orthoptera - *Schistocherca gregaria* (locust)

For each species, data was taken from several sources (detailed in Table S2 and Table S3) and used to compute the drag when streamlined, and if the insect had maintained the body angle that it used when hovering across its range of flight speeds. In many cases values were found from interpolating or extrapolating from published figures, and studies from which data has been collected span seven decades, during which technological advances may have improved measurement accuracy for some variables. Hence the data in this section aims to provide a general indication that streamlining benefits flying animals, rather than to make precise comparisons between their power requirements.

Figure 6 (main text) uses the advance ratio of each insect species as the independent variable when plotting data from multiple species on the same plot. Whilst the insects examined here span three orders of magnitude in terms of mass, and also in terms of body Reynolds number over their flight range, their advance ratios during fast forward flight all fall within the range 0.4 to 1, making this a useful measure with which to compare flight power.

The values for the advance ratio (J), Reynolds number (Re), body drag ($D*$) and parasitic power ($P*_{par}$) were calculated from measured morphological and kinematic parameters (mass (m), wing (r) and body length (l), wingbeat frequency (n), wingbeat amplitude (Φ), body angle (χ), coefficient of drag (C_d, dependent on body angle and Reynolds number) and body plan area (A)) at each flight speed (U) using the following equations[4,5]:

Advance ratio $J = U/(2.\Phi.n.R)$

Reynolds number $Re = U.l/v$ v=1.46 x 10^{-5} $m^2.s^{-1}$ (kinematic viscosity of air)

Body drag $D* = ½\ p.C_d.A.U^2/m$ p=1.23 kg.m^{-3} (density of air)

Parasitic power $P*_{par} = D*.U$

Both parasitic power and body drag are expressed relative to the insect's body mass, *m*, as such they are labeled P^*_{par} and D^* respectively. The values for all parameters are shown in the following two tables, with the exception of the coefficient of drag, body plan areas, and parasitic power. Some sources provided drag measurements and not values for the coefficient of drag or body plan areas, hence we include only the value for body drag, whether calculated or taken directly from a source. The values for parasitic power are plotted in Figure 6 (main text).

Table S2: Morphological details. The superscripted number denotes the source of data, and superscripted letters denote a footnote following the table. Values separate by commas are for the forewings and hindwings respectively.

Insect	Mass (mg) - *m*	Body length (mm) - *l*	Wing length (mm) - *r*
Apis	100 [4]	14.2 [4]	9.5 [4]
Bombus	175 [6]	18.6 [6]	13.5 [6]
Manduca	1199 [7]	45.6 [7]	47.3 [7]
Trypoxylus	5050 [8]	40.0 [8]	50.0 [8]
Sympetrum	127 [9]	35.4 [9]	27.3, 26.5 [9]
Drosophila	2 [10]	3.3 [r]	3.0 [10]
Schistocherca	1850 [11]	46.0 [11]	48.5, 44.4 [11]

[r] Assuming a wing to body length ratio of 1.1 as for Drosophila melanogaster[4]

Table S3: Flight kinematics and power requirement details. The superscripted number denotes the source of data, and superscripted letters denote a footnote following the table. Unless followed by further superscripts, those at the lowest air speed for each species apply to the values for the following air speeds of that parameter. Drag Not-SL indicates the drag value was calculated assuming the insect had maintained the same body angle for hovering for all air speeds.

**Insect**	**Air speed (m/s) - *U***	**Advance ratio - *J***	**Reynolds number - *Re***	**Wingbeat freq. (Hz) - *n***	**Wingbeat amp. (deg) - *Φ***	**Body angle (deg) - χ**	**Drag (% mass) – *D****	**Drag NSL (% mass) – *D****
Apis	0	0	0	230 [12, h]	91 [12, h]	33 [2]	0	0
	2	0.3	1775	230	91	25	3.3 [13]	6.5 [13, e]
	3	0.4	2662	230	91	24	6.3	12.5 [e]
	4	0.6	3550	230	91	13	10.7	21.4 [e]
	5	0.7	4437	230	91	9	16.6	33.2 [e]
Bombus	0	0	0	155 [6]	116 [6]	47 [6]	0	0
	1.5	0.1	1250	145	112	32	0.2 [14]	0.2 [14]
	2.5	0.3	3125	152	125	25	2.2	3.3
	3.5	0.5	4375	148	114	19	5.9	7.3
	4.5	0.7	5625	144	103	13	10.4	13.8
Manduca	0	0.0	0	26 [15]	114 [15]	40 [15]	0	0
	1	0.2	4596	25	106	29	0.5 [7]	0.6 [7]
	2	0.4	5970	26	110	21	1.3	5.8
	3	0.7	8221	27	100	17	2.3	11.6
	4	1.0	10699	25	102	14	3.6	18.2
Trypoxylus	0	0	0	37 [16, h]	170 [16, h]	50 [17]	0	0
	1	0.1	2500	37	170	50	0.9 [17, p]	0.9 [17, p]
	2	0.2	5000	37	170	50	4.3	4.3
	3	0.3	7500	37	170	50	10.1	10.1
	4	0.4	10000	37	170	50	18.5	18.5

Insect	Air speed (m/s) - ***U***	Advance ratio - ***J***	Reynolds number - ***Re***	Wingbeat freq. (Hz) - ***n***	Wingbeat amp. (deg) - ***Φ***	Body angle (deg) - ***χ***	Drag (% mass) – ***D****	Drag N (% mass) – ***D****
Sympetrum	0.7	0.2 [fw]	1536	38 [18, fw]	80 [18, fw]	32 [19, da]	0.4 [9, da, e]	0.5 [9, da, e]
	1.5	0.4	3293	38	100	13	1.6	2.8
	2.3	0.5	5049	38	120	1	2.5	9.1
	3.2	0.7	7025	38	130	2	8.6	18.1
Drosophila	0	0	0	240 [10, h]	150 [10, h]	68 [20]	0	0
	0.5	0.13	103	240	150	52	3.1 [21]	3.6 [21, e]
	1	0.27	206	240	150	39	7.7	10.0 [e]
	1.5	0.4	309	240	150	22	12.8	24.2 [e]
	2	0.53	412	240	150	8	17.9	44.6 [e]
Schistocherca	0	0 [hw]	0	20 [11, h, hw, t]	109 [11, h, hw, t]	25 [22, t]	0	0
	1	0.3	2813	20	109	8	0.7 [11]	1.8 [11]
	2	0.6	5625	20	109	3	1.2	3.5
	3	0.9	8438	20	109	1	2.7	8.4
	4	1.2	11250	20	109	0	3.9	12.7
	5	1.5	14063	20	109	0	6.5	21.1

[da] Body angles were from *Anax parthenope julius*, a larger species of dragon fly. A body angle of 35° was assumed for hovering when calculating non-streamlined body drag, slightly less horizontal than the 32° observed for 0.7 m/s flight.

[e] Value found by extrapolating beyond bounds of plot.

[fw] Value for forewings, the hindwings have a similar measurement.

[h] Value was only found for this parameter at hover, and was assumed for other flight speeds.

[hw] Value for hindwings; the value for the forewings appears un-representatively large.

[p] Data from personal communications.

[t] Measurements are from tethered animals

7. Statistical tests

Common abbreviations used when reporting statistical tests in this document are: Sum of squares (SS), degrees of freedom (df), mean squares (MS), F-value (F), *p*-value (*p*), χ^2-value (χ^2) and epsilon (test statistic for sphericity tests, ε). The subscripted number following χ^2 values denotes the degrees of freedom, and for F values, the first and second subscripted values denote the between subjects and within subjects (error) degrees of freedom respectively.

7.a Test of effects of air speed and optic flow (data in Figure 2a and d (main text))

Two factor ANOVA was conducted with one within subjects factor (optic flow), and one between subjects factor (air speed) in SPSS using Type III sum of squares (used for all ANOVA comparisons). The number of bees included for each level of the between subjects factor were 11 (0 m/s), 11 (0.5 m/s), 11 (1 m/s), 12 (1.5 m/s), 11 (2 m/s), 12 (2.5 m/s), 12 (3 m/s), 13 (4 m/s) and 11 (5 m/s).Of the 54 factor combinations, only two combinations were found to be non-normal (3 m/s at 100 deg/s and 0 m/s at 400 deg/s) using Shapiro-Wilk's test. It is unlikely that these relatively minor deviations from normality will adversely influence an ANOVA's results.

The between subjects factors were found to be heteroscedastic at each of the within subjects factors levels (Table S4). Heteroscedasticity can result in the F-statistic being non-conservative (an increase in Type 1 error), particularly in an unbalanced design where the group with the smaller number of samples has increased variance, or when the change in group means is small[23,24]. Simulation studies suggest that Type 1 error rates are unlikely to exceed 7% for normal, but heteroscedastic data[23]. Outside of those constraints, the F-statistic for heteroscedastic data is usually conservative (an increase in Type 2 error), however performance is improved when the number of groups are large[23,24] (greater than ten). Notably, the variance of between subjects groups in our data does not appear to be directly proportional to the group mean (for example the variance at 0.5 m/s air speed is larger than 0 and 1 m/s, even though this air speed results in a reduced average abdomen angle, whilst higher air speeds have both reduced averages and variances, Figure 2d (main text)), so a transformation will not alleviate the issue[24], nor would a non-parametric test. We suggest that the effect of heteroscedasticity may make tests slightly more conservative, but will not adversely affect the conclusions we draw from our data. Note that heteroscedasticity only effects the between subjects factor (air speed), as its equivalent for within subjects factor can be corrected for.

Table S4: Leven's test of equality of variances performed on between subjects factors (by within subjects factor), for normal bees. All groups are heteroscedastic.

**Factor**	**100 deg/s**	**200 deg/s**	**300 deg/s**	**400 deg/s**	**500 deg/s**	**600 deg/s**
**Result**	$F_{8,95}$=3.42, ***p*=0.002**	$F_{8,95}$=3.18, ***p*=0.003**	$F_{8,95}$=4.00, ***p*=0.0004**	$F_{8,95}$=2.67, ***p*=0.011**	$F_{8,95}$=2.38, ***p*=0.022**	$F_{8,95}$=3.14, ***p*=0.003**

Mauchly's Test of Sphericity indicated that the assumption of sphericity was violated for this data, χ^2_{14}=542.5, p<0.000001, and as ε=0.31, Greehouse-Geisser correction was used to adjust degrees of freedom for the within subjects factor (Table S5) after conducting the ANOVA. Both main effects and the interaction are highly significant. Based on Figure 2d (main text) it appears that the interaction effect occurs because the range of group means is reduced over the within subjects factor (optic flow) at high levels of the between subjects factor (air speed).

Table S5: Repeated measures ANOVA results, for within and between subjects factors, for normal bees. All tested effects are significant. Un-adjusted degrees of freedom for the within subjects factor (not used in calculations) are shown in brackets.

**Within subjects**	**SS**	**df**	**MS**	**F**	***p***
**Optic flow (OF)**	15222.1	1.6 (5)	9819.5	141.39	**<0.000001**
**OF x AS**	42109	12.4 (40)	399.6	4.89	**0.000001**
Error(OF)	10227.4	147.3 (475)	69.5		
**Between subjects**	**SS**	**df**	**MS**	**F**	***p***
**Air speed (AS)**	55248.4	8	6906.1	6.35	**0.000001**
Error	103359.8	95	1088.0		

Post-hoc comparisons were made between main effects by computing *p*-values for each pairwise comparison. Family wise error control is provided by using the Dunn-Sidak pairwise comparison for within subjects comparisons, and the Games-Howell comparison (specifically for heteroscedastic data) for between subjects comparisons, both of which were performed. Low optic flow levels are significantly different from all others up to 400 deg/s after which optic flow is no longer significantly different from higher levels (Table S6). This indicates that a plateau, or saturation, of the response has been reached.

Table S6: *Post-hoc* comparisons of optic flow levels, for normal bees. Significant pairwise comparisons are highlighted in bold.

**Optic flow**	**200 deg/s**	**300 deg/s**	**400 deg/s**	**500 deg/s**	**600 deg/s**
**100 deg/s**	***p*<0.000001**	***p*<0.000001**	***p*<0.000001**	***p*<0.000001**	**p<0.000001**
**200 deg/s**		***p*<0.000001**	***p*<0.000001**	***p*<0.000001**	**p<0.000001**
**300 deg/s**			**p=0.00003**	***p*=0.00006**	***p*=0.0005**
**400 deg/s**				*p*=0.625	*p*=0.991
**500 deg/s**					*p*=1.000

As apparent from Figure 2d (main text), the response to air speed is more complicated, and divides itself into four groups (Table S7). **First,** a plateau or saturation is again reached at 2.5 m/s, after which air speed levels are no longer significantly different. **Second,** the 0 m/s air speed is significantly different from these air speeds in the plateau region, but not from flow rates lower than 2.5 m/s; 0 m/s represents the global minimum of the response. **Third**, the speed range 1 to 2 m/s represents an intermediate local minimum, which whilst not different from 0 m/s air speed, is only different from the highest points on the plateau (4 and 5 m/s), suggesting it represents slightly higher values than the 0 m/s minimum. **Fourth**, the 0.5 m/s air speed, as it is not significantly different from any other air speed. Figure 2d (main text) shows that at high optic flow the response at this air speed approaches the level the plateau, whilst at low optic flow levels the response is similar to the local minimum centered at 1.5 m/s. 0.5 m/s represents a local maximum although its strength of its response is largely dependent on the interaction with optic flow.

Table S7: *Post-hoc* comparisons of air speed levels, for normal bees. Significant pairwise comparisons are highlighted in bold.

**Air speed**	**0.5 m/s**	**1 m/s**	**1.5 m/s**	**2 m/s**	**2.5 m/s**	**3 m/s**	**4 m/s**	**5 m/s**
**0 m/s**	*p*=0.485	*p*=0.614	*p*=0.834	*p*=0.102	***p*=0.019**	***p*=0.012**	***p*=0.0005**	***p*=0.001**
**0.5 m/s**		*p*=0.981	*p*=0.977	*p*=1.000	*p*=0.999	*p*=0.998	*p*=0.781	*p*=0.855
**1 m/s**			*p*=1.000	*p*=0.864	*p*=0.259	*p*=0.180	***p*=0.001**	***p*=0.002**
**1.5 m/s**				*p*=0.907	*p*=0.357	*p*=0.283	***p*=0.012**	***p*=0.018**
**2 m/s**					*p*=0.830	*p*=0.736	***p*=0.011**	***p*=0.011**
**2.5 m/s**						*p*=1.000	*p*=0.803	*p*=0.899
**3 m/s**							*p*=0.808	*p*=0.905
**4 m/s**								*p*=1.00

7.b Test of effects of air speed and optic flow on antenna amputated bees (data in Figure 2b and e (main text))

Two factor ANOVA was conducted with one within subjects factor (optic flow), and one between subjects factor (air speed). The number of bees included for each level of the between subjects factor were 9 (0 m/s), 9 (0.5 m/s), 8 (1.5 m/s) and 12 (3 m/s). Of the 24 factor combinations none were found to be non-normal (using Shapiro-Wilk's test). However, as for the normal bees, between subjects factors were found to be heteroscedastic at most of the within subjects factors levels (Table S8). The effect of heteroscedasticity for this data is harder to predict. Heteroscedasticity is not as severe as for normal bees, with five of the size within subjects factor levels now displaying non-equal variances, which have a mean *p*-value of 0.017, over twice the mean (0.007) for the six heteroscedastic within subjects factor levels for normal bees. From simulation studies[23], it still seems unlikely Type 1 error will noticeably exceed 5%, although the test of effects for the between subjects factor may be reduced in power.

Table S8: Leven's test of equality of variances performed on between subjects factors (by within subjects factor), for antenna amputated bees. The heteroscedastic groups are highlighted in bold.

Factor	100 deg/s	200 deg/s	300 deg/s	400 deg/s	500 deg/s	600 deg/s
**Result**	$F_{3,34}$=2.75, p=0.058	$F_{3,34}$=4.17, **p=0.013**	$F_{3,34}$=3.08, **p=0.041**	$F_{3,34}$=4.18, **p=0.013**	$F_{3,34}$=4.55, **p=0.009**	$F_{3,34}$=4.43, **p=0.010**

Mauchly's Test of Sphericity indicated that the assumption of sphericity was violated for this data, χ^2_{14}=196.8, p<0.000001, and as ε=0.31, Greehouse-Geisser correction was used to adjust degrees of freedom for the within subjects factor (Table S9). The main effect of the within subjects factor (optic flow) is highly significant, and the interaction term is not significant. The between subjects factor (air speed) remains significant, but not strongly so, although the power of the test (and thus the strength of the statistical conclusion) is likely reduced due to heteroscedasticity as discussed previously. Nonetheless, it is reasonable to conclude that air speed has less effect on these bees with their amputated antenna than their un-manipulated compatriots (Section 7.a).

Table S9: Repeated measures ANOVA results, for within and between subjects factors, for antenna amputated bees. Both main effects are significant. Un-adjusted degrees of freedom for the within subjects factor (not used in calculations) are shown in brackets.

**Within subjects**	**SS**	**df**	**MS**	**F**	***p***
**Optic flow (OF)**	4632.2	1.5 (5)	3011.3	52.04	**<0.000001**
**OF x AS**	300.2	4.6 (15)	65.1	1.12	0.358
Error(OF)	3026.5	52.3 (170)	89.0		
**Between subjects**	**SS**	**df**	**MS**	**F**	***p***
**Air speed (AS)**	4152.1	3	1384.0	3.11	**0.039**
Error	15129.8	34	445.0		

Post-hoc comparisons are made between main effects by computing *p*-values for each pairwise comparison, using Dunn-Sidak and Games-Howell tests were performed for within and between subjects comparisons respectively. The response to optic flow for antenna amputated bees plateaus (Table S10) in a similar way to bees with normal antennas (Section 7.a), except that the plateau level now occurs at 300 deg/s rather than 400 deg/s. When comparing the effects of air speed (Table S11), it now appears to follow a similar plateau or saturation response to the optic flow component. Only the 0 m/s air speed condition is significantly different from the highest air speed tested (3 m/s). The local minimum at 1.5 m/s does not occur, as the response at this point is now close to the plateau, and the response at 0.5 m/s is now generally between the response for 0 and 1.5 m/s (Figure 2e (main text)), without the obvious interaction with optic flow observed for normal bees.

Table S10: *Post-hoc* comparisons of optic flow levels, for antenna amputated bees. Significant pairwise comparisons are highlighted in bold.

**Optic flow**	**200 deg/s**	**300 deg/s**	**400 deg/s**	**500 deg/s**	**600 deg/s**
**100 deg/s**	***p*=0.000001**	***p* <0.000001**	***p* <0.000001**	***p* <0.000001**	***p*<0.000001**
**200 deg/s**		***p* <0.000001**	***p*=0.000003**	***p*=0.000008**	***p*=0.000008**
**300 deg/s**			*p*=0.919	*p*=0.863	*p*=0.989
**400 deg/s**				*p*=0.997	*p*=1.000
**500 deg/s**					*p*=1.000

Table S11: *Post-hoc* comparisons of air speed levels, for antenna amputated bees. The significant pairwise comparison is highlighted in bold.

Air speed	0.5 m/s	1.5 m/s	3 m/s
**0 m/s**	p=0.658	p=0.299	**p=0.049**
**0.5 m/s**		p=0.968	p=0.554
**1.5 m/s**			p=0.693

7.c Test of effects of air speed and optic flow on antenna waxed bees (data in Figure 2c and f (main text))

Two factor ANOVA was conducted with one within subjects factor (optic flow), and one between subjects factor (air speed). The number of bees included for each level of the between subjects factor were 12 (0 m/s), 6 (0.5 m/s), 12 (1.5 m/s) and 7 (3 m/s). Of the 24 factor combinations one was found to be non-normal (0 m/s at 100 deg/s), using Shapiro-Wilk's test. As for previous comparisons, between subjects factors were found to be heteroscedastic at most of the within subjects factors levels (Table S12). Heteroscedasticity is further reduced from the previous comparisons, with only four of the six within subjects factor levels now displaying non-equal variances. It still seems unlikely Type 1 error will noticeably exceed 5%, although the test of effects for the between subjects factor may be somewhat reduced in power.

Table S12: Leven's test of equality of variances performed on between subjects factors (by within subjects factor), for antenna waxed bees. The heteroscedastic groups are highlighted in bold.

Factor	100 deg/s	200 deg/s	300 deg/s	400 deg/s	500 deg/s	600 deg/s
**Result**	$F_{3,33}$=0.78, p=0.512	$F_{3,33}$=2.42, p=0.084	$F_{3,33}$=4.29, **p=0.012**	$F_{3,33}$=4.12, **p=0.014**	$F_{3,33}$=5.05, **p=0.005**	$F_{3,33}$=5.67, **p=0.003**

Mauchly's Test of Sphericity indicated that the assumption of sphericity was violated for this data, χ^2_{14}=305.8, p<0.000001, and as ε=0.23, Greehouse-Geisser correction was used to adjust degrees of freedom for the within subjects factor (Table S13). The main effect of the within subjects factor (optic flow) is highly significant, and the interaction and between subjects factor (air speed) are both insignificant. This shows that waxing the antenna has removed variability due to varying air speed.

Table S13: Repeated measures ANOVA results, for within and between subjects factors, for antenna waxed bees. The main effect of optic flow is significant. Un-adjusted degrees of freedom for the within subjects factor (not used in calculations) are shown in brackets.

**Within subjects**	**SS**	**df**	**MS**	**F**	***p***
**Optic flow (OF)**	8184.8	1.1 (5)	7158.3	52.49	**<0.000001**
**OF x AS**	196.1	3.4 (15)	57.2	0.42	0.766
Error(OF)	5145.7	37.7 (165)	136.4		
**Between subjects**	**SS**	**df**	**MS**	**F**	***p***
**Air speed (AS)**	1628.7	3	542.9	0.72	0.546
Error	24802.1	33	751.6		

Post-hoc comparisons are made between main effects by computing *p*-values for each pairwise comparison, using the Dunn-Sidak test for within subjects comparisons. The response to optic flow for antenna amputated bees plateaus (Table S14) in a similar way to bees with normal antennas (Section 7.a), except that the plateau level now occurs at 500 deg/s rather than 400 deg/s.

Table S14: *Post-hoc* comparisons of optic flow, for antenna waxed bees. Significant pairwise comparisons are highlighted in bold.

**Optic flow**	**200 deg/s**	**300 deg/s**	**400 deg/s**	**500 deg/s**	**600 deg/s**
**100 deg/s**	***p*=0.00002**	***p*=0.000001**	***p*<0.000001**	***p*<0.000001**	***p*<0.000001**
**200 deg/s**		***p*<0.000001**	***p*<0.000001**	***p*<0.000001**	***p*<0.000001**
**300 deg/s**			***p*=0.0004**	***p*=0.00003**	***p*=0.000002**
**400 deg/s**				***p*=0.047**	***p*=0.020**
**500 deg/s**					*p*=0.726

7.d Test of effect of antennal manipulation (Figure 5 (main text))

Two factor ANOVA was conducted with one within subjects factor (optic flow), and one between subjects factor (antennal manipulation) at each of the air speeds 0, 0.5, 1.5 and m/s. In this comparison we are not particularly interested in the effect of optic flow, as its effect has already been investigated for each antennal manipulation, but include it as a main effect to permit the use of repeated measures analysis. The number of subjects and normality tests are presented in the preceding three sections, and whilst several groups do not have normal distributions, this should not affect the outcome of our analysis. Likewise, between subjects groups

are heteroscedastic, which will make our ANOVA more conservative, and non-spherical, which is corrected for using Greehouse-Geisser correction.

The main effect of the within subjects factor (optic flow) is highly significant at all air speeds, and the interaction effect with antenna manipulation is only significant at 3 m/s (Table S15). Figure 2f (main text) shows that the range of response to optic flow increases when the antenna is manipulated at this air speed, accounting for the significant interaction at that air speed. The between subjects factor (antenna manipulation), is only significant at 0 and 1.5 m/s, not 0.5 or 3 m/s. The antennae must provide information to regulate abdomen at those two positions.

Table S15: Repeated measures ANOVA results, for within and between subjects factors at different air speeds. Significant effects are shown in bold. Un-adjusted degrees of freedom for the within subjects factor (not used in calculations) are shown in brackets.

**0 m/s**	**Within subjects**	**SS**	**df**	**MS**	**F**	***p***
	**Optic flow (OF)**	9591.9	1.4 (5)	7074.9	56.06	**<0.000001**
	**OF x AM**	818.1	2.7 (10)	301.7	2.39	0.089
	Error(OF)	4961.6	39.3 (145)	126.2		
	**Between subjects**	**SS**	**df**	**MS**	**F**	***p***
	**Antenna Manipulation (AM)**	16466.5	2	8233.3	10.43	**0.0004**
	Error	22887.0	29	789.2		
**0.5 m/s**	**Within subjects**	**SS**	**df**	**MS**	**F**	***p***
	**OF**	6436.8	1.5 (5)	4181.9	40.41	**<0.000001**
	**OF x AM**	601.7	3.1 (10	195.4	1.88	0.148
	Error(OF)	3663.7	35.4 (115)	103.5		
	**Between subjects**	**SS**	**df**	**MS**	**F**	***p***
	**AM**	1109.9	2	555.0	0.29	0.752
	Error	44178.8	23	1920.8		

1.5 m/s	**Within subjects**	**SS**	**df**	**MS**	**F**	***p***
	**OF**	5262.6	1.4 (5)	3829.3	40.87	**<0.000001**
	**OF x AM**	259.7	2.7 (10)	94.5	1.01	0.394
	Error(OF)	3734.1	39.9 (145)	93.7		
	**Between subjects**	**SS**	**df**	**MS**	**F**	***p***
	**AM**	6104.3	2	3052.1	41.19	**0.030**
	Error	22291.1	29	768.7	3.97	
**3 m/s**	**Within subjects**	**SS**	**df**	**MS**	**F**	***p***
	**OF**	2615.8	2.0 (5)	1303.6	64.70	**<0.000001**
	**OF x AM**	210.4	4.0 (10)	52.4	2.60	**0.045**
	Error(OF)	1132.1	56.2 (140)	20.2		
	**Between subjects**	**SS**	**df**	**MS**	**F**	***p***
	**AM**	1907.9	2	953.9	1.12	0.339
	Error	23735.4	28	847.7		

Post-hoc tests were conducted using least significant difference tests. These tests do not provide Type 1 error control, but as we are testing are pre-existing hypothesis that manipulated bees would be different from normal bees, rather than a full set of pairwise comparisons family wise error rate control is not necessary[25]. Significant differences between normal bees and both antennal manipulations occur at 0 and 1.5 m/s (Table S16).

Table S16: *Post-hoc* comparisons of normal antenna to both manipulations. Significant pairwise comparisons are highlighted in bold. Pairwise comparisons were performed regardless of the significance of main effects to provide consistency cross air speeds.

**Air speed**	**Normal vs. antenna amputated**	**Normal vs. antenna waxed**
**0 m/s**	***p*=0.029**	***p*=0.00008**
**0.5 m/s**	*p*=0.972	*p*=0.502
**1.5 m/s**	***p*=0.050**	***p*=0.013**
**3 m/s**	*p*=0.937	*p*=0.176

7.e Test of effect of wind on decapitated bees (Section 2)

One factor ANOVA was conducted with a single between subjects factor (air speed). Seven honeybee bodies were tested at all air speed levels. Of the 5 factor levels one was found to be slightly non-normal (4 m/s) using Shapiro-Wilk's test.

Levene's test of equality of variances indicated that the assumption of homoscedasticity was not violated for this data ($F_{4,30}$=0.17, p=0.954). The main effect of the between subjects factor (air speed), is not significant (Table S17), indicating that air speeds does not significantly affect the abdomen position of decapitated bees.

Table S17: ANOVA results from SPSS, for decapitated bees. The main effect of air speed is not significant. The values tabulated are the sum of squares (SS), degrees of freedom (df), mean squares (MS), F-value (F), and *p*-value (*p*).

	**SS**	**df**	**MS**	**F**	***p***
**Air speed (AS)**	206.4	4	51.6	0.83	0.517
Error	1866.3	30	62.2		

7.f Test of effects of negative air speed (tail wind) and optic flow (Section 1)

Two factor ANOVA was conducted with one within subjects factor (optic flow), and one between subjects factor (air speed). The number of bees included for each level of the between subjects factor were 7 (0 m/s), 10 (-0.5 m/s), 9 (-1.5 m/s) and 10 (-3 m/s). Of the 24 factor combinations one was found to be non-normal (-1.5 m/s at 300 deg/s) using Shapiro-Wilk's test. The between subjects factors were found to be slightly heteroscedastic at only one of the within subjects factors levels (Table S18), and it is unlikely this would have affected the results of the ANOVA.

Table S18: Leven's test of equality of variances performed on between subjects factors (by within subjects factor), for bees experiencing a tail wind. The heteroscedastic group is highlighted in bold.

**Factor**	**100 deg/s**	**200 deg/s**	**300 deg/s**	**400 deg/s**	**500 deg/s**	**600 deg/s**
**Result**	$F_{3,32}$=0.73, p=0.541	$F_{3,32}$=0.50, p=0.683	$F_{3,32}$=3.4, **p=0.030**	$F_{3,32}$=1.47, p=0.242	$F_{3,32}$=0.67, p=0.574	$F_{3,32}$=1.3, p=0.291

Mauchly's Test of Sphericity indicated that the assumption of sphericity was violated for this data, χ^2_{14}=145.93, p<0.000001, and as ε=0.33, Greehouse-Geisser correction was used to adjust degrees of freedom for the within subjects factor (Table S19). The main effect of the within subjects factor (optic flow) is highly significant, and the between subjects factor (air speed) is weakly significant, and the interaction term is not.

Table S19: Repeated measures ANOVA results, for within and between subjects factors, for bees experiencing a tail wind. The main effects of optic flow and air speed are significant. Un-adjusted degrees of freedom for the within subjects factor (not used in calculations) are shown in brackets.

**Within subjects**	**SS**	**df**	**MS**	**F**	***p***
**Optic flow (OF)**	12170.0	1.7 (5)	7316.4	52.11	**<0.000001**
**OF x AS**	341.9	5.0 (15)	68.5	0.49	0.783
Error(OF)	7473.4	53.2 (16)	140.4		
**Between subjects**	**SS**	**df**	**MS**	**F**	***p***
**Air speed (AS)**	6267.6	3	2089.2	3.10	**0.041**
Error	21583.8	32	675.5		

Post-hoc comparisons are made between main effects by computing p-values for each pairwise comparison, using the Dunn-Sidak test for both main effects comparison. The response to optic flow for bees experiencing a tail wind (Table S20) plateaus in the same way to bees experiencing forwards air flow (Section 7.a), at 400 deg/s. The only significant difference in pairwise test of air speeds occurs between -0.5 and -1.5 m/s (Table S21). These air speeds have the largest difference in abdomen pitch responses (Figure S1b), and it appears that the response at 0.5 m/s may be slightly elevated, as for bees experiencing forwards air speed (Figure 2d (main text)).

Table S20: *Post-hoc* comparisons of optic flow, for bees experiencing a tail wind. Significant pairwise comparisons are highlighted in bold.

**Optic flow**	**200 deg/s**	**300 deg/s**	**400 deg/s**	**500 deg/s**	**600 deg/s**
**100 deg/s**	***p*=0.000001**	***p* <0.000001**	***p* <0.000001**	***p* <0.000001**	***p*<0.000001**
**200 deg/s**		***p*=0.0001**	***p*=0.000007**	***p*=0.000004**	***p*=0.00003**
**300 deg/s**			***p*=0.001**	***p*=0.040**	***p*=0.012**
**400 deg/s**				*p*=0.991	*p*=0.980
**500 deg/s**					*p*=1.000

Table S21: *Post-hoc* comparisons of air speed, for bees experiencing a tail wind. The significant pairwise comparison is highlighted in bold.

**Air speed**	**-0.5 m/s**	**-1.5 m/s**	**-3 m/s**
**-0 m/s**	*p*=0.727	*p*=0.740	*p*=0.973
**-0.5 m/s**		***p*=0.044**	*p*=0.157
**-1.5 m/s**			*p*=0.991

7.g Test of effect of optic flow presentation order (Section 4.b)

Three factor ANOVA was conducted with two within subjects factors (optic flow and order) and one between subjects factor (air speed). The number of bees included for each level of the between subjects factor were 11 (0 m/s), 12 (1.5 m/s) and 12 (3 m/s). Bees were exposed to a triangular function of optic flow levels, as described in Section 4.b. The 600 deg/s air speed point is not included in analysis as, being at the peak point of the triangular optic flow ramp; bees were not exposed to this value twice in the same flight.

For these exploratory data analyses, we elected to include flights from bees that stopped flying briefly during the stimulation protocol. Hence the missing values for four bees (missing at most two data points), were replaced with the mean of value measured for that bee over all other factor combinations. Of the 30 factor combinations three were found to be non-normal (0 m/s at 100 deg/s, first and second presentations; 0 m/s at 300 deg/s, first presentation) using Shapiro-Wilk’s test. Interestingly, between subjects factors were found to be slightly heteroscedastic at only one of the within subjects factors levels (Table S22).

Table S22: Leven's test of equality of variances on between subjects factors (by within subject factors). The heteroscedastic group is highlighted in bold.

**Presentation**			**First**		
**Factor**	**100 deg/s**	**200 deg/s**	**300 deg/s**	**400 deg/s**	**500 deg/s**
**Result**	$F_{2,32}$=0.81, p=0.455	$F_{2,32}$=0.0, p=0.997	$F_{2,32}$=1.0, p=0.370	$F_{2,32}$=0.2, p=0.816	$F_{2,32}$=0.1, p=0.913
**Presentation**			**Second**		
**Factor**	**100 deg/s**	**200 deg/s**	**300 deg/s**	**400 deg/s**	**500 deg/s**
**Result**	$F_{2,32}$=9.3, ***p*=0.001**	$F_{2,32}$=1.2, p=0.319	$F_{2,32}$=0.2, p=0.862	$F_{2,32}$=0.4, p=0.673	$F_{2,32}$=0.4, p=0.696

Table S23: Mauchly's Test of Sphericity for within subjects factors. Significant violations of the assumption of sphericity are highlighted in bold. Order has only two factor levels and is not tested.

**Effect**	χ^2	**df**	***p***	***ε***
**Optic flow (OF)**	139.1	9	**<0.000001**	0.32
**Order**	0	0	-	-
**OF x Order**	113.1	9	**<0.000001**	0.37

Mauchly's Test of Sphericity showed that the assumption of sphericity was violated for the main effect of optic flow and its interaction with presentation order (Table S23), hence, Greehouse-Geisser correction was used to adjust degrees of freedom for those within subject factors in the following ANOVA (Table S24). Consistent with the results observed for normal bees (Section 7.a), the main effects of optic flow and air speed are both significant, although interestingly the interaction between the two is not significant (although this is likely because the reduction in air speed levels tested has reduced the power of this ANOVA test to detect this interaction relative to others in this paper, rather than the effect itself having changed). The order of presentation of optic flow has no significant main effect, or significant interactions associated with any other factors. This indicates that the abdomen position is relatively independent on the order of optic flow presentation, at least in the case of a simple triangular function.

Table S24: Repeated measures ANOVA results. The significant effects are highlighted in bold. Un-adjusted degrees of freedom for within subjects factors (not used in calculations) are shown in brackets.

**Within subjects factors**	**SS**	**df**	**MS**	**F**	***p***
**Optic flow (OF)**	7312.8	1.3 (4)	5732.4	22.81	**0.000005**
**OF x AS**	1402.2	2.6 (8)	549.6	2.19	0.113
Error(OF)	10259.2	40.8 (128)	251.3		
**Order**	6.7	1	6.8	0.10	0.750
**AS x Order**	316.1	2	158.0	2.41	0.106
Error(Order)	2095.2	32	65.5		
**OF x Order**	34.1	1.5 (4)	23.0	0.19	0.759
**OF x Order x Wind**	250.8	3.0 (8)	84.5	0.71	0.551
Error(OFxOrder)	5668.7	47.5 (128)	119.4		
**Between subjects factor**	**SS**	**df**	**MS**	**F**	***p***
**Air speed (AS)**	18473.2	2	9236.6	8.79	**0.001**
Error	33627.7	32	1050.9		

7.h Test of effect of air speed presentation order (Section 4.a)

Three factor ANOVA was conducted with three within subjects factors (optic flow, air speed and order). Ten bees participated in all experiments. Bees were exposed to a triangular function of air speed levels, as described in Section 4.a. The 5 m/s air speed point is not included in analysis as, being at the peak point of the triangular air speed ramp, bees were not exposed to this value twice in the same flight.

For these exploratory data analyses, we elected to include flights from bees that stopped briefly flying during the stimulation protocol. Hence the missing values for three bees (missing for at most two data points), were replaced with the mean value measured for that bee at all other factor combinations. Of the 24 factor combinations two were found to be non-normal (100 deg/s at 0 m/s, first presentation; and 500 deg/s at 0 m/s, first presentation) using Shapiro-Wilk's test.

Table S25: Mauchly's Test of Sphericity for within subjects factors. Significant violations of the assumption of sphericity are highlighted in bold. Order has only two factor levels and is not tested.

**Effect**	χ^2	**df**	***p***	***ε***
**Optic flow (OF)**	3.5	2	0.175	0.74
**Air speed (AS)**	19.2	9	**0.027**	0.44
**Order**	0	0	-	-
**OF x AS**	107.6	35	**<0.0000001**	0.30
**OF x Order**	0.9	2	0.957	0.99
**AS x Order**	33.7	9	**0.0001**	0.40
**OF x AS x Order**	109.5	35	**<0.0000001**	0.25

Mauchly's Test of Sphericity showed that the assumption of sphericity was violated for some main effects and interactions (Table S25). The Greehouse-Geisser correction was used to adjust degrees of freedom for those factors in the following ANOVA (Table S26). The main effects of optic flow and air speed are significant for this hysteresis test, and they show a significant interaction. The effect of air speed presentation order is not significant itself, however, it does show a significant interaction with air speed, and additionally shows a significant three factor interaction with the optic flow and air speed. As we might expect, optic flow does not interact significantly with the order of air speed presentation. Thus, it appears that presentation order of air speeds affects how honeybees respond to that stimulus and modifies how air speed interacts with optic flow, resulting in the hysteresis curves observed for triangular air speed ramps across all tested optic flow speeds (Section 4.a).

Table S26: Results of repeated measures ANOVA. The significant main effects and interactions are highlighted in bold. Un-adjusted degrees of freedom for the within subjects factors (not used in calculations) are shown in brackets.

**Within subjects factors**	**SS**	**df**	**MS**	**F**	***p***
**Optic flow (OF)**	839.6	2	419.8	5.46	**0.014**
Error(OF)	1384.3	18	76.9		
**Air speed (AS)**	8970.1	1.8 (4)	5122.0	64.41	**<0.000001**
Error(AS)	1253.4	15.8 (36)	79.5		
**Order**	48.0	1	48.0	0.76	0.407
Error(Order)	572.7	9	63.6		
**OF x AS**	1101.7	2.4 (8)	463.5	5.81	**0.007**
Error(OFxAS)	1706.6	21.4 (72)	79.8		
**OF x Order**	54.6	2	27.3	0.74	0.493
Error(OFxOrder)	668.4	18	37.1		
**AS x Order**	1250.1	1.6 (4)	780.7	11.44	**0.002**
Error(ASxOrder)	983.8	14.4 (36)	68.3		
**OF x AS x Order**	685.5	2.0 (8)	348.2	5.41	**0.015**
Error(OFxASxOrder)	1140.1	17.7 (72)	64.3		

7.i Test of effect of air speed and optic flow when the thorax is pitched up (Section 3)

Two factor ANOVA was conducted with one within subjects factor (optic flow) and one between subjects factor (air speed) using data for bees tethered with their thorax pitched up. The number of bees included for each level of the between subjects factor were 10 (0 m/s), 8 (1.5 m/s) and 10 (3 m/s). Of the 18 factor combinations for each level of the thorax pitch factor, none were non-normal (Shapiro-Wilk's test). Whilst the between subjects factor was heteroscedastic at most of the within subjects factors levels (Table S27), as for bees tethered with their thorax horizontal (Section 7.a), we suggest that this will make our ANOVA conservative.

Table S27: Leven’s test of equality of variances performed on between subjects factors (by within subjects factor), for bees with their thorax pitched up. All bar one group are heteroscedastic.

**Factor**	**100 deg/s**	**200 deg/s**	**300 deg/s**	**400 deg/s**	**500 deg/s**	**600 deg/s**
**Result**	$F_{2,25}$=17.04, ***p*=0.00002**	$F_{2,25}$=5.00, ***p*=0.015**	$F_{2,25}$=7.10, ***p*=0.004**	$F_{2,25}$=4.16, ***p*=0.028**	$F_{2,25}$=2.59, *p*=0.095	$F_{2,25}$=6.65, ***p*=0.005**

Mauchly’s Test of Sphericity indicated that the assumption of sphericity was violated for this data, χ^2_{14}=130.35, p<0.000001, and as ε=0.33, the Greehouse-Geisser correction was used to adjust degrees of freedom for the within subjects factor (Table S28). Both main effects, optic flow and air speed, including their interaction, are significant, matching our findings for bees with their thorax tethered horizontally (Section 7.a), and suggesting their behavior is qualitatively similar.

Table S28: Repeated measures ANOVA results, for within and between subjects factors, for bees with their thorax pitched up. All effects are significant. Un-adjusted degrees of freedom for the within subjects factor (not used in calculations) are shown in brackets.

**Within subjects**	**SS**	**df**	**MS**	**F**	***p***
**Optic flow (OF)**	7222.2	1.6 (5)	4403.9	28.31	**<0.000001**
**OF x AS**	2290.0	3.3 (10)	698.2	4.49	**0.007**
Error(OF)	6378.6	41.0 (125)	155.6		
**Between subjects**	**SS**	**df**	**MS**	**F**	***p***
**Air speed (AS)**	20781.0	2	10390.5	11.10	**0.0004**
Error	23396.2	25	935.8		

Whilst bees appear to react in a similar manner to both stimuli when their thorax is pitched up, to make direct comparisons we conducted three factor ANOVA, with an additional between subjects factor, the thorax pitch. Data from Figure 2(a and d (main text)) for bees with a horizontal thorax (at 0, 1.5 and 3 m/s air speeds) and those with a pitched up thorax comprised the two levels of the new factor. The number of subjects, normality and equality of variance tests for bees with a horizontal thorax are listed previously in Section 7.a. Whilst heteroscedasticity is not corrected for, the Greehouse-Geisser correction is used for within-subjects factors. Air speed, optic flow and their interaction are significant, whereas the thorax angle does not have a significant main effect or interactions (Table S29). This confirms that bees with their thoraxes pitched up exhibit a streamlining response to optic flow and air speed that is statistically indistinguishable from those with their thorax horizontal.

Table S29: Repeated measures ANOVA results. The significant effects are highlighted in bold. Un-adjusted degrees of freedom for within subjects factors (not used in calculations) are shown in brackets.

**Within subjects factors**	**SS**	**df**	**MS**	**F**	***p***
**Optic flow (OF)**	13941.3	1.7 (5)	8002.3	82.46	**<0.000001**
**OF x AS**	3929.4	3.5 (10)	1127.7	11.62	**<0.000001**
**OF x Angle**	208.7	1.7 (5)	119.8	1.24	0.293
**OF x AS x Angle**	297.7	3.5 (10)	85.4	0.88	0.467
Error(OF)	9636.5	99.3 (285)	97.0		
**Between subjects factor**	**SS**	**df**	**MS**	**F**	***p***
**Air speed (AS)**	40374.4	2	20187.2	18.6	**0.000001**
**Angle**	188.6	1	188.6	0.17	0.678
**AS x Angle**	149.1	2	74.6	0.07	0.934
Error(AS)	61735.8	57	1083.1		

7.j Test of effect of air speed and optic flow when the thorax is pitched down (Section 3)

Two factor ANOVA was conducted with one within subjects factors (optic flow) and one between subjects factors (air speed) using data for bees tethered with their thorax pitched down. The number of bees included for each level of the between subjects factor were 5 (0 m/s), 7 (1.5 m/s) and 7 (3 m/s).

For these exploratory data analyses, we elected to include flights from bees that stopped flying briefly during the stimulation protocol. Hence the missing values for three bees (missing at most one data point), were replaced with the mean value measured for that bee at all other factor combinations. Of the 18 factor combinations for each level of the thorax pitch factor, two were found to be non-normal (3 m/s at 500 deg/s and 1.5 m/s at 600 deg/s), using Shapiro-Wilk's test. Whilst the between subjects factor was heteroscedastic at half of the within subjects factors levels (Table S30), as for bees tethered with their thorax horizontal (Section 7.a), we suggest this will make our ANOVA slightly conservative.

Table S30: Leven's test of equality of variances performed on between subjects factors (by within subjects factor), for bees with their thorax pitched down. Half of the groups are heteroscedastic.

Factor	100 deg/s	200 deg/s	300 deg/s	400 deg/s	500 deg/s	600 deg/s
**Result**	$F_{2,16}$=2.24, p=0.139	$F_{2,16}$=2.18, p=0.146	$F_{2,16}$=2.39, p=0.123	$F_{2,16}$=5.76, **p=0.013**	$F_{2,16}$=7.06, **p=0.006**	$F_{2,16}$=4.74, **p=0.024**

The Mauchly's Test of Sphericity indicated that the assumption of sphericity was violated for this data, χ^2_{14}=37.08, p<0.001, and as ε=0.59, the Greehouse-Geisser correction was used to adjust degrees of freedom for the within subjects factor (Table S31). The main effect of air speed, including its interaction with optic flow, is significant. Surprisingly, the main effect of optic flow was not significant, in contradiction to the findings for bees with their thoraxes tethered horizontally (Section 7.a). Whilst this test has a reduced number of subjects compared relative to the previous ones (due to the insects reluctance to fly when tethered with their thorax down), which would have reduced its power, the data in Section 3 shows that bees tethered with their thorax down have a qualitatively different reaction to optic flow. This suggests that tethering bees with thoraxes pitched down changed their behavior, and indeed changed the effect optic flow has on regulating their abdomen position. Referees

Table S31: Repeated measures ANOVA results, for within and between subjects factors, for bees with their thorax pitched downwards. Un-adjusted degrees of freedom for the within subjects factor (not used in calculations) are shown in brackets.

**Within subjects**	**SS**	**df**	**MS**	**F**	***p***
**Optic flow (OF)**	369.5	2.93 (5)	125.8	1.58	0.208
**OF x AS**	1310.8	5.9 (10)	223.2	2.80	**0.021**
Error(OF)	3743.4	47.0 (80)	79.7		
**Between subjects**	**SS**	**df**	**MS**	**F**	***p***
**Air speed (AS)**	13098.4	2	6549.2	8.19	**0.004**
Error	12794.3	16	799.6		

8. Supplementary references

1 Luu, T., Cheung, A., Ball, D. & Srinivasan, M. V. Honeybee flight: a novel 'streamlining' response. *J. Exp. Biol.* **214**, 2215-2225 (2011).

2 Nachtigall, W., Widmann, R. & Renner, M. Über den «ortsfesten» freien Flug von Bienen in einem Saugkanal. *Apidologie* **2**, 271-282 (1971).

3 Lin, S. NGPM -- A NSGA-II program in Matlab v1.4. *MATLAB Central File Exchange.* (2011).

4 Ellington, C. P. The aerodynamics of hovering insect flight. 2. Morphological parameters. *Phil. Trans. R. Soc. B: Biol. Sci.* **305**, 17-40 (1984).

5 Ellington, C. P. The aerodynamics of hovering insect flight. 3. Kinematics. *Phil. Trans. R. Soc. B: Biol. Sci.* **305**, 41-78 (1984).

6 Dudley, R. & Ellington, C. P. Mechanics of forward flight in bumblebees. 1. Kinematics and morphology. *J. Exp. Biol.* **148**, 19-52 (1990).

7 Willmott, A. P. & Ellington, C. P. The mechanics of flight in the hawkmoth *Manduca sexta*. II. Aerodynamic consequences of kinematic and morphological variation. *J. Exp. Biol.* **200**, 2723-2745 (1997).

8 McCullough, E. L., Weingarden, P. R. & Emlen, D. J. Costs of elaborate weapons in a rhinoceros beetle: how difficult is it to fly with a big horn? *Behav. Ecol.* **23**, 1042-1048 (2012).

9 Wakeling, J. M. & Ellington, C. P. Dragonfly flight. I. Gliding flight and steady-state aerodynamic forces. *J. Exp. Biol.* **200**, 543-556 (1997).

10 Weis-Fogh, T. Energetics of hovering flight in hummingbirds and in *Drosophila*. *J. Exp. Biol.* **56**, 79-104 (1972).

11 Weis-Fogh, T. Biology and physics of locust flight. II. Flight performance of the desert locust (*Schistocerca gregaria*). *Phil. Trans. R. Soc. B: Biol. Sci.* **239**, 459-510 (1956).

12 Altshuler, D. L., Dickson, W. B., Vance, J. T., Roberts, S. P. & Dickinson, M. H. Short-amplitude high-frequency wing strokes determine the aerodynamics of honeybee flight. *Proc. Natl. Acad. Sci. USA* **102**, 18213-18218 (2005).

13 Nachtigall, W. & Hanauer-Thieser, U. Flight of the honeybee. 5. Drag and lift coefficients of the bee's body; implications for flight dynamics. *J. Comp. Physiol. B* **162**, 267-277 (1992).

14 Dudley, R. & Ellington, C. P. Mechanics of forward flight in bumblebees. 2. Quasi-steady lift and power requirements. *J. Exp. Biol.* **148**, 53-88 (1990).

15 Willmott, A. P. & Ellington, C. P. The mechanics of flight in the hawkmoth *Manduca sexta*. I. Kinematics of hovering and forward flight. *J. Exp. Biol.* **200**, 2705-2722 (1997).

16 Van Truong, T., Le, T. Q., Byun, D., Park, H. C. & Kim, M. Flexible wing kinematics of a free-flying beetle (Rhinoceros beetle *Trypoxylus dichotomus*). *J. Bionic Eng.* **9**, 177-184 (2012).

17 McCullough, E. L. & Tobalske, B. W. Elaborate horns in a giant rhinoceros beetle incur negligible aerodynamic costs. *Proc. R. Soc. B: Biol. Sci.* **280** (2013).

18 Wakeling, J. M. & Ellington, C. P. Dragonfly flight. II. Velocities, accelerations and kinematics of flapping flight. *J. Exp. Biol.* **200**, 557-582 (1997).

19 Azuma, A. & Watanabe, T. Flight performance of a dragonfly. *J. Exp. Biol.* **137**, 221-252 (1988).

20 Sun, M. & Wu, J. H. Aerodynamic force generation and power requirements in forward flight in a fruit fly with modeled wing motion. *J. Exp. Biol.* **206**, 3065-3083 (2003).

21 Vogel, S. Flight in *Drosophila.* I. Flight performance of tethered flies. *J. Exp. Biol.* **44**, 567-578 (1966).

22 Camhi, J. M. Sensory control of abdomen posture in flying locusts. *J. Exp. Biol.* **52**, 533-537 (1970).

23 Bao, P. & Ananda, M. M. A. Performance of two-way ANOVA procedures when cell frequencies and variances are unequal. *Comm. in Stat. - Sim. and Comp.* **30**, 805-829 (2001).

24 Krutchkoff, R. G. One—way fixed effects analysis of variance when the error varianes may be unequal. *J. Stat. Comp. and Sim.* **30**, 259-271 (1988).

25 Ruxton, G. D. & Beauchamp, G. Time for some a priori thinking about post hoc testing. *Behav. Ecol.* **19**, 690-693 (2008).
